# Applications of the FIV Model to Study HIV Pathogenesis

**DOI:** 10.3390/v10040206

**Published:** 2018-04-20

**Authors:** Craig Miller, Zaid Abdo, Aaron Ericsson, John Elder, Sue VandeWoude

**Affiliations:** 1 Department of Veterinary Pathobiology, Oklahoma State University, Stillwater, OK 74078, USA; 2 Department of Microbiology, Immunology, and Pathology, Colorado State University, Fort Collins, CO 80523, USA; Zaid.Abdo@colostate.edu (Z.A.); Sue.Vandewoude@colostate.edu (S.V.); 3 Department of Veterinary Pathobiology, University of Missouri, Columbia, MO 65201, USA; EricssonA@missouri.edu; 4 Department of Immunology and Microbiology, The Scripps Research Institute, La Jolla, CA 92037, USA; jelder@scripps.edu

**Keywords:** feline immunodeficiency virus, FIV, human immunodeficiency virus, HIV, animal models, opportunistic disease, lentiviral pathogenesis, molecular biology

## Abstract

Feline immunodeficiency virus (FIV) is a naturally-occurring retrovirus that infects domestic and non-domestic feline species, producing progressive immune depletion that results in an acquired immunodeficiency syndrome (AIDS). Much has been learned about FIV since it was first described in 1987, particularly in regard to its application as a model to study the closely related lentivirus, human immunodeficiency virus (HIV). In particular, FIV and HIV share remarkable structure and sequence organization, utilize parallel modes of receptor-mediated entry, and result in a similar spectrum of immunodeficiency-related diseases due to analogous modes of immune dysfunction. This review summarizes current knowledge of FIV infection kinetics and the mechanisms of immune dysfunction in relation to opportunistic disease, specifically in regard to studying HIV pathogenesis. Furthermore, we present data that highlight changes in the oral microbiota and oral immune system during FIV infection, and outline the potential for the feline model of oral AIDS manifestations to elucidate pathogenic mechanisms of HIV-induced oral disease. Finally, we discuss advances in molecular biology, vaccine development, neurologic dysfunction, and the ability to apply pharmacologic interventions and sophisticated imaging technologies to study experimental and naturally occurring FIV, which provide an excellent, but often overlooked, resource for advancing therapies and the management of HIV/AIDS.

## 1. Feline Immunodeficiency Virus

Feline immunodeficiency virus (FIV) is a naturally-occurring retrovirus that infects domestic and non-domestic feline species. In domestic cats, FIV produces progressive immune depletion that eventually results in an acquired immunodeficiency syndrome (AIDS) [1,2,3,4,5,6,7,8,9,10]. As a consequence, FIV infection is associated with a variety of clinical syndromes, including cachexia, anterior uveitis, chronic rhinitis, gingivostomatitis and periodontitis, encephalitis and neurologic dysfunction, and lymphoma [1,4,9,11,12,13,14,15,16,17,18,19,20,21]. The acute phase of FIV infection, lasting approximately 4–8 weeks, is characterized by a sharp increase in CD4+ T lymphocytes that are accompanied by high levels of FIV viral RNA and proviral DNA in circulation [4,8,22]. These hematologic changes are typically accompanied by mild to moderate clinical signs, which include pyrexia, lethargy, and peripheral lymphadenopathy [4,22,23]. Following a prolonged asymptomatic phase, during which the levels of circulating virus remains stable and integrated provirus establishes a reservoir of latently infected target cells, there is progressive decline of CD4+ T lymphocytes and other immunocytes, resulting in functional immunodeficiency and susceptibility to opportunistic infections [6,24,25,26].

During FIV infection, the loss of CD4+ T lymphocytes is directly attributable to a viral-induced cytopathic effect, in addition to an increase in FIV-specific CD8-mediated programmed cell death, lack of thymic regeneration, and spontaneous apoptosis in response to decreased cytokine support [10,25,27,28]. The most frequent clinical disease syndromes that are associated with FIV infection manifest as a consequence of immune dysfunction, such as oral opportunistic infection (gingivitis, stomatitis, and periodontitis), immune-mediated glomerulonephritis, chronic rhinitis, and dermatitis [15,16,19,20,29,30]. Oral opportunistic infections are prevalent in a high proportion of FIV-infected cats, and frequently present as erythematous, inflammatory lesions along the gingival margin (gingivitis), multifocal areas of necrotizing inflammation within the gingival sulcus or periodontal ligament (periodontitis), or ulcerative inflammatory lesions along the buccal mucosa, hard palate, or soft palate (stomatitis) [20,31,32,33]. Changes in the salivary/oral microbiota have been increasingly associated with FIV infection, and shifts in the proportion of opportunistic pathogens in the saliva of FIV-infected cats have been associated with the development of oral inflammatory lesions [33,34]. Similarly, FIV-infected cats frequently present with severe, necrotizing, and/or ulcerative inflammatory lesions (dermatitis) due to opportunistic infection with various bacterial, fungal, protozoal, and parasitic etiologies, including mycobacteriosis, leishmaniasis, toxoplasmosis, and dermatophytosis [16,29,35,36]. Upper respiratory disease is also a frequently finding in FIV-infected cats, and may occur in conjunction with concurrent viral, bacterial, or fungal infections [4,15,37,38].

**Figure 1:**
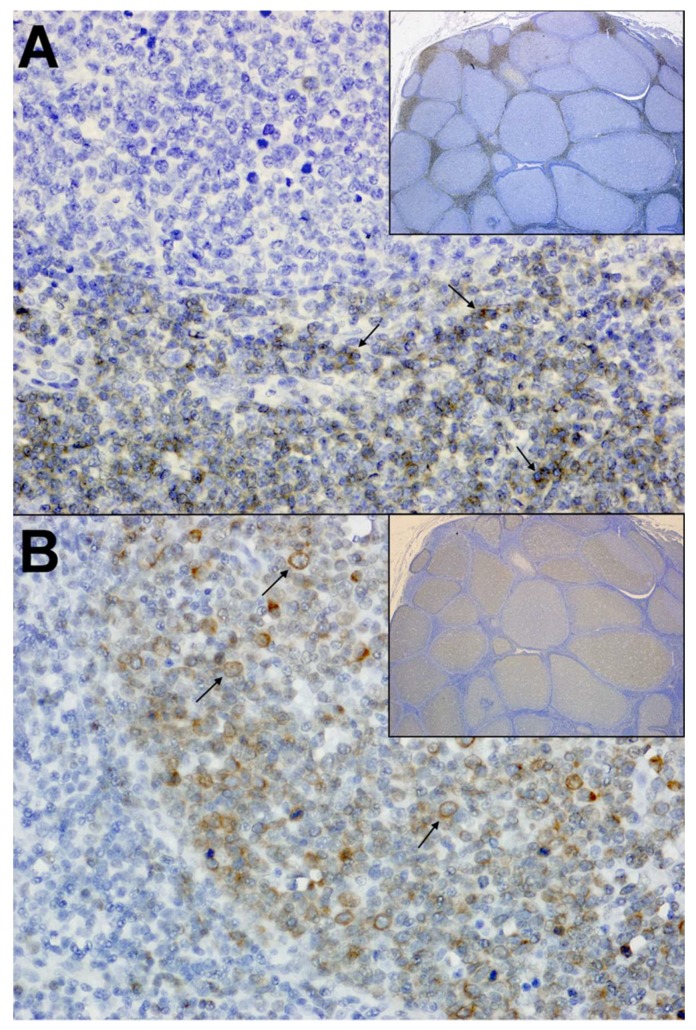
Immunohistochemistry from a feline immunodeficiency virus (FIV)-infected cat with primary B cell lymphoma. (**A**) Mesenteric lymph Node; 40× and 4× (inset). Normal amounts of interlobular T lymphocytes (arrows) are present throughout the lymph node. Anti-CD3 (IHC) with DAB as chromogen and hematoxylin counterstain. (**B**) Mesenteric lymph Node; 40× and 4× (inset). Neoplastic B lymphocytes (arrows) multifocally expand the normal lymph node architecture. Anti-CD79a IHC with DAB as chromogen and hematoxylin counterstain.

Interestingly, FIV is also associated with the occurrence of neoplastic diseases, most frequently being demonstrated by the development of lymphoma in a large proportion of infected cats [7,39]. This association has been described in both naturally and experimentally infected animals, and predominately manifests as high-grade B-cell neoplasms that are remarkably similar to HIV-associated diffuse large B-cell lymphoma (DLBCL) (Figure 1) [7,40,41,42]. Also similar to HIV, direct viral-mediated oncogenesis that is related to proviral integration within oncogenes is an uncommon feature of FIV infection, and neoplastic transformation has been attributed to the indirect consequences of viral-induced immune dysfunction that arise in response to prolonged viral infection [7,42,43,44]. Specifically, recent studies have shown that clonal proviral integration sites are not typically detected during FIV infection and that proviral loads are lower in neoplastic tissues, indicating the neoplastic growth of cells lacking provirus [7]. Conversely, FIV and other lentiviral infections are strongly associated with polyclonal B-cell expansion, immunoglobulin production, and cytokine expression of proliferative mediators in response to immune activation and dysregulation [45,46]. It is proposed that such infection kinetics provide opportunities for somatic rearrangements that are associated with the generation of B-cell receptor diversity, or mutations in immunological cells during rapid expansion that disrupt or activate oncogenes; thus, resulting in neoplastic transformation [7,42]. However, the causal relationship of FIV and lymphoma has not been fully elucidated, and further studies are necessary to evaluate the specific role that viral infection and immune function play during tumorigenesis.

FIV-induced renal disease is also observed in both experimentally and naturally infected cats, and includes pathologic changes that include glomerulonephritis, proteinuria, protein tubular casts, and tubular microcysts, as well as diffuse interstitial inflammatory infiltrates [30,47]. Mesangial widening with glomerular and interstitial amyloidosis is also observed in kidneys of FIV-infected cats, and when evaluated in the context of another frequent finding during FIV infection, hypergammaglobulinemia, indicate the potential for immune complex deposition to occur within the glomerulus as a result of chronic antigenic stimulation and immune activation [30,48,49].

Neurologic disease is an important manifestation of FIV infection, and affected cats may present with either central nervous system (CNS) or peripheral nervous system (PNS) involvement [14,17,18,50,51]. In the PNS, FIV induces significantly increased the numbers of CD3+ T cells and macrophages in dorsal root ganglia, and infected cats exhibit pronounced changes in epidermal nerve fiber densities [50,52]. FIV enters the CNS during the acute stages of infection and it is present within the brain and cerebral spinal fluid [14,17,53]. The primary neuropathogenic effect of FIV infection within the CNS manifests as infiltration and accumulation of perivascular lymphocytes and macrophages (encephalitis), activation of microglial cells and astrocytes (gliosis), and occasional neuronal loss with myelin degeneration [14,17,18,53,54]. This infiltration of inflammatory cells and the consequences that are associated with immune activation within the CNS frequently results in clinically apparent neurologic deficits and a gradual decline in CNS function, functionally manifesting as abnormal stereotypic motor behaviors, anisocoria, increased aggression, prolonged latencies in brainstem evoked potentials, delayed righting and pupillary reflexes, decreased nerve conduction velocities, and deficits in cognitive-motor functions [55,56,57,58].

## 2. FIV as a Molecular Analogue to HIV

FIV is a member of the Lentivirus genus within the Retroviridae family, and much has been learned about FIV since it was first described in 1987, particularly in regard to its application as a model to study the closely related lentivirus, human immunodeficiency virus (HIV) [8,9,10,59,60]. The FIV virion is approximately 100 nm in diameter, spherical, and contains two identical strands of positive-sense RNA in its 9400-base genome, which is tightly associated with the nucleocapsid protein (NC, p7) and a t-RNA^lys^ bound to each RNA molecule, which serves as a primer for negative strand transcription [25,59,60,61]. This protein complex, along with viral enzymes that are involved with replication and maturation (protease, reverse transcriptase, integrase, and dUTPase), are enclosed within a core of capsid protein (CA, p24), and are surrounded by a shell of matrix protein (MA, p14) [25,59,60]. Viral envelope glycoproteins (gp) are embedded within an outer lipid bilayer surrounding the matrix coat, and include the surface (SU, gp95) and transmembrane (TM, gp40) subunits, which are cleaved from a 130–150 kDa membrane-bound precursor protein, glycosylated, and non-covalently anchored within the envelope in a trimeric form [25,59,60,62].

**Figure 2:**
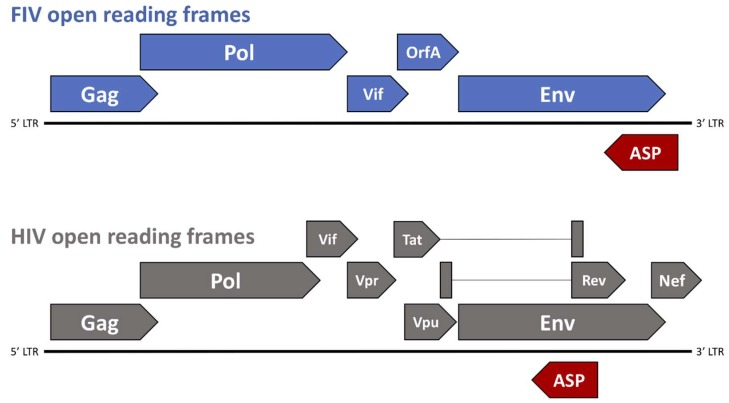
Genomic organization of FIV and human immunodeficiency virus (HIV). The genomic structure of FIV consists of three primary open reading frames (ORFs), *gag*, *pol*, *and env*, which are flanked by two long-terminal repeats (LTR) and accompanied by numerous small ORFs containing regulatory and accessory genes such as *vif* and *orfA.* Potential short ORFs (antisense ORFs—ASP, shown in red) may be translated from a negative strand message. The genomic structure of HIV consists of three similar primary ORFs (*gag*, *pol*, and *env*), as well as numerous small ORFs containing regulatory and accessory genes, such as *vif*, *vpr*, *vpu*, *tat*, *rev*, and *nef.* Similar to FIV, the antisense ORF, ASP (shown in red) may be translated from a negative strand message.

The genomic structure of FIV consists of three primary open reading frames (ORFs), *gag*, *pol*, and *env*, which are flanked by two long-terminal repeats (LTR) and are accompanied by numerous small ORFs containing regulatory and accessory genes, such as *vif*, *rev*, and *orfA* (Figure 2). FIV *gag* encodes the Gag polyprotein, which is cleaved by the protease to form the three mature proteins (MA, CA, and NC) and is necessary to achieve formation of mature virus particles [59,63,64]. Pol polyprotein, which is the primary product of the FIV *pol* gene, contains four important enzymes that are involved in virus replication and maturation: protease, reverse transcriptase (RT), integrase (IN), and dUTPase (DU) [59]. Viral protease (PR) facilitates the cleavage of Gag and Pol polyproteins into functional enzymatic or structural proteins; DU catalyzes the hydrolysis of dUTP to dUMP in effort to minimize misincorporation of potentially mutagenic dUTP into host DNA [59,65,66]; FIV RT is an RNA-dependent DNA polymerase involved in the reverse transcription of viral genomic RNA into a double-stranded copy of proviral DNA (cDNA). Once synthesized, cDNA is integrated into the host genome by a mature IN containing three functional domains: an N-terminal domain, a central catalytic core, and a C-terminal domain [67,68,69]. The FIV Env polyprotein, which is a 130–150 kDa product of the *env* gene, is glycosylated and trimmed within the Golgi apparatus, and proteolytically cleaved into two mature, glycosylated proteins prior to virion budding at the cell surface: SU (gp95) and TM (gp40), both of which play critical roles in virion attachment and entry into target cells [59,60].

The structural and sequence organization of FIV is very similar to HIV, which is also a member of the lentivirus genus [59]. HIV is morphologically characterized by a spherical virion that is roughly 120nm in diameter, and contains a diploid genome that is composed of two copies of single stranded, positive-sense RNA that is packaged with nucleocapsid (p7) and accessory proteins (protease, reverse transcriptase, integrase) [70]. Like FIV, the ribonucleoprotein complex at the heart of the HIV virion is contained within a dense core of Capsid protein (CA, p24) and is surrounded by a spherical shell of Matrix protein (MA, p17) [70]. Mature Env glycoproteins, SU (gp120), and TM (gp 41), are anchored within the external lipid bilayer, and play a significant role in cell entry through binding to host cell receptors.

FIV requires an initial interaction with a primary binding receptor for infection, and binds to host cells through a high-affinity interaction of the envelope SU protein (gp95) with the CD134 surface molecule present on CD4+ lymphocytes and monocytes/macrophages [71,72,73,74,75]. This interaction induces a conformational change in the SU protein, which then exposes a cryptic epitope in the V3 loop of Env; the binding site that is necessary for binding with the entry (co-) receptor CXCR4 [26,74,75]. Binding of the V3 loop exposes the serpentine region of TM (gp40), which results in the formation of a hairpin structure that allows the fusion with the cell membrane and subsequent cell entry [26,75,76]. However, as infection progresses, the production of neutralizing antibodies by the host increases the need for FIV to escape selective pressures. New viral variants arise that exhibit a decreased dependence on CD134 and an increased ability to infect cells that express CXCR4 with limited CD134 expression, such as naïve B cells, macrophages, and CD8+ T cells [2,3,60,77,78,79,80]. This expanded cell tropism results in an increase in the number of target cells that are susceptible to infection, which subsequently causes immunodepletion and clinical manifestations that are associated with AIDS-induced disease.

HIV also requires an initial interaction with a primary binding receptor for infection, and utilizes analogous modes of receptor-mediated entry as FIV utilizing chemokine co-receptors [81,82,83]. However, in lieu of CD134, HIV utilizes CD4 as the primary binding receptor and CCR5 as its primary entry receptor, although HIV is also able to utilize CXCR4 [81,82]. Much like FIV, HIV binds to CD4+ target cells through a high-affinity interaction with the CD4 receptor that induces a conformational change in the envelope glycoprotein gp120, subsequently exposing the binding sites necessary for chemokine co-receptor binding (CXCR4 or CCR5) and subsequent fusion with the cell membrane. In HIV infection, the expression of CCR5 or CXCR4 chemokine receptors on target cells is the primary determinant of cell tropism, with CCR5-mediated infection of CD4+ T cells predominating early in infection [84,85,86]. However, over the course of infection, the preference of HIV for CCR5 co-receptor usage changes to CXCR4 in up to half of infected individuals, and these CXCR4-utilizing strains exhibit a broader tropism for different T-cell subpopulations [84,87]. Furthermore, differences in CD4 and CCR5 expression levels can affect the CCR5-mediated infection of macrophages, resulting in a shift in cell tropism that is similar to what is observed during FIV infection [84,86,87].

The HIV genome encodes three primary polyproteins, Gag, Pol, and Env, as well as the regulatory protein, Rev, and accessory protein, Vif—all of which exhibit similar functions to FIV [59,60,70]. However, in addition to these, HIV also contains genes that encode additional accessory proteins that are involved in viral maturation, replication, and survival [70]. These accessory proteins include: Tat (p16/p14), a viral transcriptional activator; Vpr (p10–15), a promoter of nuclear localization and inhibitor of cell division (cell cycle arrest at G2/M); Vpu (p16); a promotor of extracellular release of viral particles; Nef (p27–25), a downregulator of CD4 and MHC I expression; Vpx (p12–16), a Vpr homolog present in HIV-2 (absent in HIV-1); and, Tev (p28), a tripartite tat-env-rev protein [70].

The FIV genome contains one regulatory gene (*rev*) and two accessory genes (*vif* and *orfA*). FIV *rev* encodes Rev, which is a nucleolar polyprotein that binds to the Rev Response Element (RRE) to allow for export of partially spliced and unspliced viral RNA transcripts out of the nucleus with the help of the nuclear export protein, exportin-1 [59,60,88]. The FIV Vif protein is crucial to FIV replication and is involved in the counteraction of host defense mechanisms, such as APOBEC3, which is a cellular protein that exerts an antiviral effect by deamination of cytosine to uracil during viral replication, resulting in the degradation of synthesized minus-strand DNA [59,60,89]. FIV Vif counteracts APOBEC3 by targeting the host protein to the E3 ubiquitin ligase complex, which is subsequently degraded by the proteasome [59,60,89].

The FIV OrfA protein is encoded by the accessory gene *orfA* (Figure 2), and was originally considered to be a transactivator of transcription due to a role in increasing the net translation of proteins that were expressed from genes under the transcriptional control of the FIV LTR. The localization of the *orfA* gene in the viral genome also roughly coincides with the location of the gene encoding the HIV transactivator, Tat [90]. However, studies have failed to show an increase in transcription directed by OrfA, and there is no trans-activation response (TAR) element, as acted on by HIV Tat. Thus, an increase in net protein translation that is facilitated by OrfA must be by other means and may be involved in late steps of virion formation and the early steps of virus infectivity, although the precise role of OrfA is still undetermined [59,60,91,92,93,94]. OrfA localizes in the nucleus and causes cell cycle arrest at G2 in infected cells, reminiscent of effects that are caused by the Vpr protein in HIV-1. Also, OrfA has been shown to downregulate expression of the viral receptor for FIV (CD134) on the surface of cells, as well as E2 ubiquitin-conjugating enzymes and an ubiquitin-protein ligase [60,90,95], which is similar to the effects that are ascribed to the Nef protein on CD4 downregulation during HIV-1 infection. These potential functions of OrfA may have implications that aid in viral dissemination by preventing surface interactions with budding virions, and limit the degradation of viral proteins by host cell ubiquitin ligase mechanisms.

In 1988, Miller [96] made the observation that there was also potential to encode a peptide product from an RNA transcribed from the minus strand of the provirus. Since then, there have been a number of reports providing evidence for predicted RNA and protein products from the minus strand in HIV-1 [97,98,99,100,101,102,103,104,105,106], SIV [107], FIV [108], and in the deltaretrovirus, BLV [109]. In FIV, there are several potential short open reading frames that may be translated from a negative strand message. However, the major potential reading frame in the negative strand of both FIV and HIV, antisense protein (ASP), coincides with the Env coding region in the plus strand RNA, in the region underlying the Rev Responsive Element (RRE) encoded on the plus strand (Figure 2). A recombinant protein that has been transcribed and translated from the ASP open reading frame has been used to screen both naturally and experimentally FIV infected cats for antibodies to the protein and a small percentage (<10%) do show some level of positivity (manuscript in preparation) (Figure 2). Furthermore, knocking out the putative start codon for ASP resulted in a dramatic reduction in viral protein production, suggesting a critical role in the virus life cycle. Immunohistochemistry shows a non-nuclear localization of the protein, which is suggestive of some post-transcription event. Further studies will be required to define the role of ASP, but it may contribute to the ability of the virus to replicate by counteracting some innate anti-viral response in the cell.

## 3. FIV as a Model to Study HIV Pathogenesis

### 3.1. Immune Dysfunction

The primary immunodeficiency of FIV, which is a gradual and progressive decline in CD4+ T lymphocytes, is a hallmark feature of both natural and experimental infection, and the most obvious fundamental feature to parallel HIV infection. During both FIV and HIV infection, CD4+ lymphocyte numbers decline over an extended asymptomatic phase, and are associated with an increase in activated CD8+ lymphocytes that have antiviral activity [110,111,112,113]. The net effect of this event is a decrease in the ratio of CD4+ cells to CD8+ cells (CD4:CD8), and is used as a clinical indicator of immunosuppression in both FIV and HIV infected patients [112,113,114]. Additionally, several studies have shown that FIV induces defects in immune function that are similar to HIV, such as a decreased proliferation response of T lymphocytes in response to mitogens, a deficit in the humoral immune response, and the dysregulation of cytokine expression [10,24,59].

**Figure 3:**
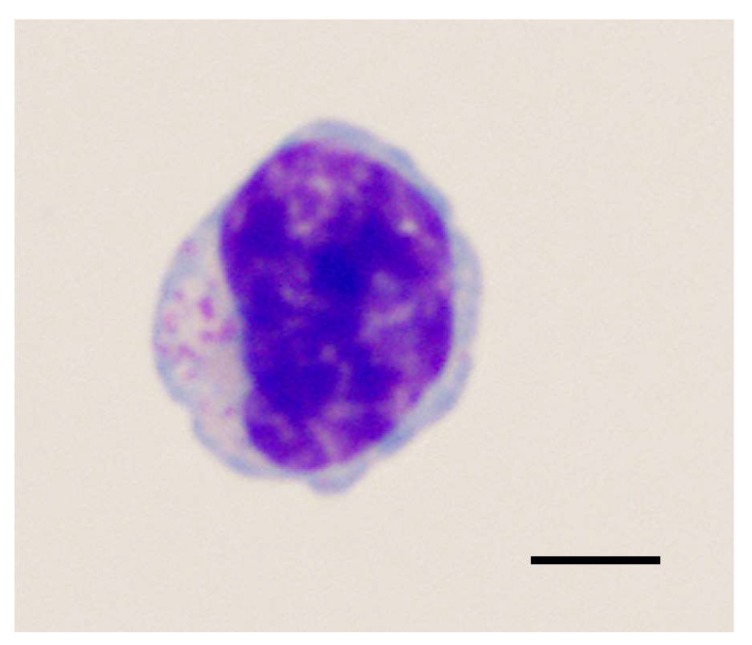
Cytological morphology of a large granular lymphocyte (LGL). Recent studies have determined that elevations in LGL phenotypes during both FIV and HIV infection may represent polyclonal T-cells with viral suppressive properties. Wright-Giemsa stain, bar = 5 μm.

Large granular lymphocytes (LGLs) are a lymphoid subset comprising 10–15% of peripheral mononuclear blood cells (PBMCs) (Figure 3), and consist of either CD3− NK cells or CD3+ T-cells that mediate antibody-dependent cytotoxicity [115,116,117,118]. Analysis of LGL populations during HIV infection have been hampered by the low percentage of these cells in circulation, and has typically only been reported in association with neoplasia [118,119,120]. However, recent studies have shown that LGLs are detectable and are elevated during HIV infection in humans, and may represent viral-suppressive CD8+ T cells [118,121]. Interestingly, studies in FIV-infected cats have determined that similar elevations in LGL phenotypes may represent polyclonal T-cells with viral suppressive properties, as indicated by increased interferon-γ (IFN-γ) expression and decreased PBMC proviral loads in correlation with LGL lymphocytosis [118,122].

Conversely, recent studies have shown that CD4+ CD25+ T regulatory (Treg) cells are responsible for the inhibition of CD8+ IFN-γ production during both FIV infection [123] and HIV infection [124], highlighting the potential mechanisms by which these viruses exhibit an immunosuppressive effect on the CD8+ immune response. Furthermore, additional studies have shown that FIV directly infects and activates CD4+ CD25+ Treg cells, which are then able to suppress CD4+ CD25− T helper (Th) cells [125]. While this relationship and the potential mechanisms of Treg cell activation during HIV infection is still unclear, such comparative studies in FIV may offer potential to help our understanding of CD8+ T cell function in HIV infection.

### 3.2. Neurologic Dysfunction

Previous studies have shown that both FIV and HIV enter the central nervous system (CNS) at acute stages of infection, either via the trafficking of infected monocytes and lymphocytes, or by the penetration of free virus across the blood-brain or blood-CSF barriers [17,126,127,128,129,130,131]. Once present in the CNS, both FIV and HIV infection spread to microglia and astrocytes, which then serve as a reservoir for latent viral persistence [13,17,130,131,132]. Although multinucleated giant cells are rarely observed in the CNS during FIV infection, the fundamental neuropathologic finding of encephalitis is well-documented in both HIV and FIV infected patients, and the resultant proliferation and activation of these cells (gliosis) is associated with neurodegenerative processes, such as myelin degradation and neuronal injury/loss [14,17,51,54,133]. Thus, the clinical manifestations that are associated with neuropathology of FIV are likewise observed in HIV infection, and because of this, FIV has been repeatedly used as a model to investigate the pathogenesis of dementia and cognitive-motor processing deficits in AIDS patients. In vitro models of FIV have been useful to expand our understanding of role of calcium dysregulation and neural dysfunction during lentiviral infection, and have provided a unique system for the development neuroprotective treatments, such as neurotrophin ligands, which prevent the delayed accumulation of intracellular calcium and the decreased cytoskeletal damage of neuronal dendrites [17,134]. Furthermore, because of the low natural prevalence and the slow clinical course that is associated with lentiviral-induced neurologic dysfunction, experimental in vivo studies have been developed in the FIV model, which accelerate neuropathogenesis (neonatal inoculation, inoculation with neurovirulent strains, direct intracranial inoculation), allowing for increased opportunity to evaluate the viral kinetics of CNS infection, neurovirulence determinants, and the potential for novel treatments that are designed to decrease neurocognitive defects during HIV infection [53,57,134,135].

The use of neurovirulent strains of FIV has also allowed for the investigation of neuropathogenic effects on the peripheral nervous system (PNS) as a model of HIV distal symmetric polyneuropathy (DSP), demonstrating the rapid onset of peripheral neuropathy in FIV infected cats with axonal injury, macrophage activation, and detection of the virus within the nerve [50,136]. Indeed, FIV infection results in pathological events in the PNS that are very similar to HIV, including increased numbers of CD3+ T lymphocytes and activated macrophages in skin and dorsal root ganglia (DRGs) that are associated with increased expression of the pro-inflammatory cytokines, as well as changes in epidermal nerve fiber densities, which is indicative of axonal and myelin degeneration [50,52]. FIV has also been useful in the evaluation of the neurotoxicity of antiretroviral toxic neuropathy (ATN), due to mitochondrial dysfunction that is associated with nucleoside analogue reverse transcriptase (NRTI) inhibitor treatment. Thus, FIV has the potential to expand our understanding of the role of the immunopathology and progression of neuropathy in FIV-infected cats.

SIV models of neuropathogenesis have been used to study HIV-associated neurologic dysfunction (HAND), and have resulted in the elucidation of many mechanisms of neuroAIDS development, such as acute CNS infection and the importance of monocyte/macrophage activation in driving CNS lesions [137,138,139,140]. Recently, the SIV model of neuroAIDS has been adapted to study peripheral neuropathy, and significant advances have been made that implicate macrophages within dorsal root and trigeminal ganglia as a source of viral maintenance, in addition to their role in neuronal loss and neuronophagia [141,142]. These findings are coupled with additional studies that have defined impaired mitochondrial function in distal axons, which are more pronounced in ART-treated animals, indicating the potential for antiretroviral-mediated mitochondrial toxicity [143]. However, the SIV model of HAND is most commonly employed in rhesus macaques using SIV strains that arose via nosocomial infections or a lab adaptation of African monkey strains [144]. SIV neurologic disease is therefore chiefly manifested as a rapid progression to AIDS with the hallmarks of CNS inflammation that amplify pathology when compared to HIV-infected humans [139,140]. Furthermore, NHP studies are also limited by increased zoonotic risk to researchers, high cost associated with animal care and housing, the low number of animals available for research, and the potential for co-infection with a wide array of other pathogens, including rhesus rhadinovirus (RRV), lymphocryptovirus (LCV), simian cytomegalovirus (CMV), simian foamy virus (SFV), simian virus 40 (SV40), and rhesus papillomavirus (RhPV) [145,146].

In mechanistic studies of HIV-associated neurologic dysfunction, the interaction of CXCR4 with viral envelope has been shown to enhance neuronal apoptosis via Ca^2+^-regulating systems and NMDA receptors (NMDARs) in the synaptic membrane [147,148,149,150,151,152,153]. This neurotoxic pathway is known to involve Ca^2+^ influx through NMDARs, nitric oxide (NO) production, and subsequent activation cGMP-dependent protein kinase II, however, the precise cellular mechanisms by which this occurs are unknown and are difficult to assess in chronically infected human patients [154,155,156,157,158,159]. Because FIV binds to CXCR4 on the neuronal membrane in a similar non-infectious interaction as HIV, the feline model may provide answers, particularly in regard to the viral envelope-receptor interaction and synaptic activity-mediated neurotoxicity in HAND [160,161]. Given these similarities (and limitations of the SIV model), FIV represents an adjunct lentiviral model that can accurately recapitulate neuroAIDS progression in HIV-infected humans for applications, such as evaluation of ART-induced neurotoxicity, neurofibrillary tangle development, and calcium homeostasis during viral infection [14,17].

### 3.3. Vaccine Development

Considerable effort has been directed at the development of an anti-HIV vaccine strategy that can produce protective immunity in humans, and this effort has been paralleled in regard to FIV. A commercially available, whole inactivated virus vaccine containing two FIV subtypes (Fel-O-Vax FIV^®^) is currently licensed for use in the United States, and various reports have described virus neutralization and cellular immunity in a significant proportion of study animals [162,163,164]. However, the efficacy of this vaccine is still under debate, as recent studies and field evaluations have reported that the vaccine does not confer immunity against certain FIV strains (i.e., FIV_GL8_), and that the neutralizing antibody response and the protective rate may be low in certain cat populations (i.e., protection is not conferred to certain virulent recombinant strains of FIV) [165,166,167,168]. Other attempts at FIV vaccine development have either failed to induce protective immunity against FIV infection, or have resulted in an increased susceptibility to infection via antibody-dependent enhancement or general immune activation [169,170,171,172,173,174].

The development of an anti-HIV vaccine has been impeded by a wide variety of similar complications, such as lack of efficacy or unanticipated side effects, as well as increased susceptibility to infection via the analogous mechanisms of FIV vaccine enhancement (antibody-dependent viral enhancement or general immune activation) [175,176,177,178,179,180,181]. Indeed, vaccine-induced enhancement of viral infection has been previously reported in a large number of HIV studies [182,183,184,185], and it has been shown to occur via antibody-dependent or antibody-independent mechanisms of complement activation [186,187,188,189,190,191,192,193], as well as an increase in general immune activation and/or the expansion of lymphoid target cells [194,195,196,197,198]; features that have also been observed in FIV studies [169,170,171,172,173,174]. However, despite these setbacks in lentiviral vaccine development, there are many similarities in the disease course of HIV and FIV infection, and the use of the FIV model to circumvent these roadblocks may have great potential to provide a translational model for the development of novel immunotherapies to protect from HIV infection in humans.

Traditionally, non-human primate (NHP) models have been at the forefront of anti-HIV vaccine development due to the similarities of SIV and HIV, and have revealed several promising vaccine targets, such as *nef*-deleted SIV (which protects from wild-type SIV infection) and broad neutralizing antibodies utilizing chimeric SHIVs that express the HIV-1 envelope glycoprotein [199,200,201,202]. However, the successful outcome of these methods to prevent HIV infection in humans has been significantly impeded by various causes, such as restrictions on the use of live-attenuated HIV-1 in humans, as well as difficulty in producing a sufficiently efficacious neutralizing antibody response by vaccination [200]. Alternatively, various humanized mouse models have played a vital role in elucidating key aspects of the immune response to HIV, primarily through the use of generally immunocompromised mice that were engrafted with reconstituted human immune system tissues, such as human fetal thymus and liver (scid-*hu-Thy/Liv*), or peripheral blood lymphocytes (scid-*hu-PBL*) [203]. These models have been used for key studies in HIV immunopathogenesis, including mechanisms of CD4+ T-cells loss, antiretroviral therapy response, and passive immunization with monoclonal antibodies to HIV envelope protein (and testing of *Env-*based vaccines) [146,203,204,205,206,207]. However, because only certain parts of the human immune system can be reconstituted in humanized mouse models, interactions between the introduced human cells and the murine immune system cannot be evaluated in these hosts, nor the effects of HIV infection in non-hematopoietic tissues [203]. Although FIV lacks certain molecular similarities to HIV, it induces similar immunopathologies in its natural host, and therefore represents an important yet underutilized animal model for fully evaluating the immune response during natural lentiviral infection. Furthermore, the availability of a commercially-available vaccine in cats with efficacy against at least a subset of FIV may provide important clues to improving the efficacy of anti-HIV vaccines, and the elucidation of the mechanisms that are associated with vaccination failure in analogous FIV and HIV models of immunotherapy may provide key insights into improving the efficacy of lentiviral vaccines.

### 3.4. HIV-Induced Oral Disease

Oral manifestations of HIV are exhibited through various disease syndromes, such as Oral Candidiasis (OC, “thrush”), Linear Gingival Erythema (LGE), Necrotizing Ulcerative Gingivitis (NUG), and Necrotizing Ulcerative Periodontitis (NUP) [208,209,210]. Despite the success of combinational antiretroviral therapy (cART) in diminishing HIV viral replication and prolonging immune function, lesions that are associated with systemic and local immune activation and opportunistic oral infections persist in HIV-infected patients [208,211,212,213]. Previous studies have demonstrated that CD4+ T-cells are rapidly and severely depleted from the intestinal mucosa following HIV infection due to the direct effects of targeted virus infection and virus-induced Fas-mediated apoptosis, resulting in a loss of mucosal integrity and a reduced capacity to control potential pathogens at mucosal surfaces—thereby triggering local and systemic pro-inflammatory responses [214,215,216,217]. Based upon the analogous microenvironments of the oral and gastrointestinal mucosa, the same effects of viral-induced immunosuppression is predicted to occur in the oral cavity, resulting in a chronic cycle of immune stimulation, leukocyte recruitment, and target cell infection that produces HIV-induced oral disease lesions [208,218].

The FIV model is particularly well suited for studies of HIV-associated oral disease, as it not only parallels HIV in its structural, biochemical, and immunological properties, but it is also the only naturally occurring lentivirus to predictably induce oral lesions in its natural host, the domestic cat [1,4,9,10,31,32]. Non-human primate (NHP) models of HIV do not reliably cause oral disease and are limited by zoonotic risk to researchers, high cost associated with animal care and housing, and low number of animals that are available for research, while humanized mouse models of HIV lack both the prevalence of oral lesions and the presence of tonsillar structures that are similar to humans [146,219,220,221]. In contrast, FIV oral manifestations are common in naturally and experimentally-infected cats [20,32,33], and the range of lesions seen include gingivitis, periodontitis, and feline chronic gingivostomatitis [32], with striking similarities to LGE, NUG, and NUP lesions noted in untreated HIV patients [1,4,111,208,222,223,224,225]. Furthermore, opportunistic infections that were detected in HIV-positive individuals are paralleled in feline oral disease syndromes [35,226,227,228,229,230,231,232,233,234,235], and feline tonsillar tissues (palatine, pharyngeal, and lingual tonsils) are analogous to those in humans [220]. Coupled with recent advances in new generation cART protocols that have potential for adaption in cat studies [236,237,238,239,240], the domestic cat model of FIV presents an easily manipulated animal model to evaluate the drivers of immune dysfunction and microbial dyscrasias during HIV infection using a controlled in vivo study design.

**Figure 4:**
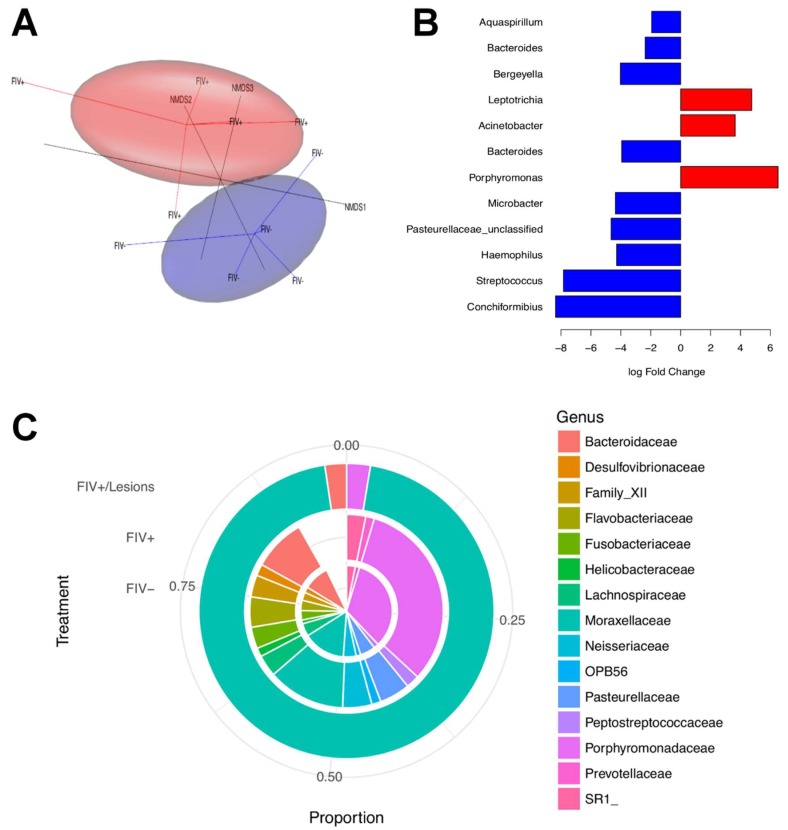
Salivary microbiome alterations during FIV infection. (**A**) Three-dimensional (3D) Nonmetric Multidimensional Scaling (NMDS) separates clusters of FIV− and FIV+ cat microbiome samples. Ovals represent the 90% confidence ellipsoids around the centroid of the clusters (FIV+ = red; FIV− = blue). (**B**) Operational taxonomic units (OTUs) with significant log-fold change in abundance between FIV+ and FIV− cats at the 0.1 level of significance (after correcting for multiple testing). The list on the left shows the genera of each of these OTUs. Red indicates over representation of that OTU in the FIV+ cats. (**C**) FIV+ cat with clinical gingivitis/periodontitis with near monoculture of *Moraxellaceae* (outer circle) as compared to the mean microbial community structure of cats that are FIV + (middle circle) and cats that are FIV negative (inner circle).

Thus, in order to assess in vivo mechanisms contributing to oral disease during lentiviral infection, we collected saliva from the sublingual area and ventral cheek pouches from juvenile SPF cats (12–14 month-old) and examined samples by 16S rRNA metagenomics analysis to detect differences in the oral microbiota of naïve and age-matched cats that were infected with FIV (PPR strain) of eight months duration (*n* = 5/group). FIV_PPR_ is a relatively apathogenic strain of FIV that typically results in mild self-limiting gingivitis and/or periodontitis during acute infection [241], and animals did not have overt, visual signs of clinical periodontitis at the time of sampling. FIV-infected and naïve SPF animals were maintained on a similar diet, and similar anatomic regions were swabbed from all of the animals at the same time of day. DNA was extracted [242], and amplicon sequencing was performed using illumina MiSeq to generate paired-end 2 × 250 bp sequences of the hyper-variable region 4 (V4) of the 16S rDNA. Data were normalized using cumulative sum scaling [243], and used to construct a nonmetric multidimensional scaling three-dimensional (3D) plot (Figure 4A).

Significant differences were detected in the oral microbiota composition of FIV-infected cats relative to naïve animals (Figure 4A). Normalized data were tested using the Zero Inflated Gaussian model implemented in the R package metagenomeSeq [244] to identify the putative OTUs driving differences between FIV+ and FIV− cats. Significant log-fold change in abundance in 12 genera was noted between groups at the 0.1 level of significance after correction for multiple testing (Figure 4B). One FIV-positive cat developed moderate to severe erythematous gingivitis during the course of infection and saliva was collected and analyzed as described above. Upon analysis of saliva, this individual demonstrated a dramatically altered microbiome population with >95% operational taxonomic units (OTUs), corresponding to the genus *Moraxellaceae* as compared to the FIV+ cats with no lesions and the FIV− cats (Figure 4C).

Collectively, these results demonstrate that similar to HIV, FIV infection of domestic cats is associated with oral microbiota dysbiosis and a marked loss of microbial diversity during lentiviral-associated periodontitis. The persistence of HIV infection and periodontitis in patients on cART indicates that ancillary treatments that are specifically directed at restoring the normal oral microbiota in conjunction with cART may improve HIV periodontal progression and decrease systemic immune activation [229,245,246,247]. Feline dental disease is currently managed by comprehensive dental treatment consisting of hand and ultrasonic scaling, identical to techniques that are used in humans [248,249]. Probiotic supplementation has been successful in early studies as an adjuvant for treating periodontitis in people, and similar commercial oral probiotics products are available for the management of feline oral conditions [250,251,252]. Thus, the application of comprehensive dental cleaning with probiotic treatments in the feline model has the potential to assess the impact of local therapy for restoring oral homeostasis during lentiviral infection, and may increase our understanding of the progression and/or resolution of FIV-induced oral lesions and oral microbiome in the presence and absence of cART.

## 4. Conclusions

Our understanding of FIV infection of cats has progressed remarkably over the last three decades, yet much remains to be learned from this widespread lentiviral infection. Correspondingly, many aspects of HIV pathogenesis and the mechanisms of immune dysfunction are still poorly understood. Most notably, the complete elimination of HIV from the host and the restoration of immune function has not yet been achieved, nor has the means to provide protective immunity from infection. In regard to the future of HIV research, a precise understanding of the mechanisms for immunodeficiency, especially in the face of co-infections, viral-associated disease, and in the presence and absence of antiretroviral therapy will be necessary for the development of restorative or immuno-protective therapies and prophylaxis.

While being genetically divergent, FIV shares remarkable overlap with HIV in regard to molecular biology and function. Coupled with the flexibility of working with a small animal model, FIV represents a useful system to assess the in vivo aspects of lentiviral pathogenesis. As noted above, comparative pathogenesis of lentiviral immune dysfunction, neurologic, and oral disease in the feline model could aid in an understanding of HIV AIDS. Further, the successful deployment of an FIV vaccine provides great opportunities for the evaluation of lentiviral prophylaxis leading to sterilizing immunity.

The application of investigations in the molecular biology and function of genetic elements is another area that affords great potential to understand the mechanisms of lentiviral infection via the FIV model. For example, contemporary studies in FIV have recently used the 3D structure of FIV reverse transcriptase to uncover the mechanistic basis of viral resistance to non-nucleoside inhibitor drugs [253]. These studies are now uncovering crucial elements in RT structure that can be used as a template for the development of novel compounds that target conventional sites of drugs resistance, providing increased efficacy against drug-resistant strains of HIV [253].

Finally, the FIV model holds significant potential as a tractable vehicle to assess the efficacy of novel anti-retroviral therapies. Recent studies employing a progressive cART regimen that is composed of nucleoside reverse transcriptase inhibitors (emtricitabine, tenofovir) and integrase inhibitors (dolutegravir) have demonstrated the significant efficacy in FIV studies in vitro [236,237,238,239,240], and immuno-restorative therapies employing recombinant feline interferon omega (rFeIFN-ω) have resulted in the improvement of clinical symptoms in FIV-associated oral disease and feline chronic gingivostomatitis [254,255,256]. IFN-ω has also been reported to be a potent inhibitor of HIV infection in vitro, but in vivo therapeutic potential in human patients has not been evaluated [257]. Because IFN-ω exerts strong immunomodulatory effects by stimulating Natural Killer cell activity, enhancing the expression of MHC-I, and inhibiting lymphocyte proliferation, testing outcomes of IFN-ω therapy on FIV-associated disease may therefore elucidate anti-inflammatory mechanisms and offer significant potential for adoption as an agent to treat HIV-associated diseases [258].

Improvements in molecular technology and available diagnostic analyses for domestic cats, as well as the ability to apply pharmacologic interventions and sophisticated imaging technologies to the study of experimental and naturally occurring FIV provide an excellent, but often overlooked resource for advancing the therapies and management of HIV/AIDS.

## References

[1] Siebelink K.H., Chu I.-H., Rimmelzwaan G.F., Weijer K., van Herwijnen R., Knell P., Egberink H.F., Bosch M.L., Osterhaus A.D. (1990). Feline immunodeficiency virus (FIV) infection in the cat as a model for HIV infection in man: Fiv-induced impairment of immune function. AIDS Res. Hum. Retrovir..

[2] Dean G.A., Himathongkham S., Sparger E.E. (1999). Differential cell tropism of feline immunodeficiency virus molecular clones in vivo. J. Virol..

[3] English R.V., Johnson C.M., Gebhard D.H., Tompkins M.B. (1993). In vivo lymphocyte tropism of feline immunodeficiency virus. J. Virol..

[4] Pedersen N., Yamamoto J.K., Ishida T., Hansen H. (1989). Feline immunodeficiency virus infection. Vet. Immunol. Immunopathol..

[5] Torten M., Franchini M., Barlough J.E., George J.W., Mozes E., Lutz H., Pedersen N.C. (1991). Progressive immune dysfunction in cats experimentally infected with feline immunodeficiency virus. J. Virol..

[6] Hosie M.J., Addie D., Belák S., Boucraut-Baralon C., Egberink H., Frymus T., Gruffydd-Jones T., Hartmann K., Lutz H., Marsilio F. (2009). Feline immunodeficiency: Abcd guidelines on prevention and management. J. Feline Med. Surg..

[7] Magden E., Miller C., MacMillan M., Bielefeldt-Ohmann H., Avery A., Quackenbush S.L., VandeWoude S. (2013). Acute virulent infection with feline immunodeficiency virus (FIV) results in lymphomagenesis via an indirect mechanism. Virology.

[8] Pedersen N.C., Ho E.W., Brown M.L., Yamamoto J.K. (1987). Isolation of a t-lymphotropic virus from domestic cats with an immunodeficiency-like syndrome. Science.

[9] Elder J.H., Lin Y.-C., Fink E., Grant C.K. (2010). Feline immunodeficiency virus (FIV) as a model for study of lentivirus infections: Parallels with HIV. Curr. HIV Res..

[10] Burkhard M., Dean G.A. (2003). Transmission and immunopathogenesis of fiv in cats as a model for HIV. Curr. HIV Res..

[11] Bęczkowski P.M., Litster A., Lin T.L., Mellor D.J., Willett B.J., Hosie M.J. (2015). Contrasting clinical outcomes in two cohorts of cats naturally infected with feline immunodeficiency virus (FIV). Vet. Microbiol..

[12] Colitz C.M. (2005). Feline uveitis: Diagnosis and treatment. Clin. Tech. Small Anim. Pract..

[13] Dow S.W., Poss M.L., Hoover E.A. (1990). Feline immunodeficiency virus: A neurotropic lentivirus. J. Acquir. Immune Defic. Syndr..

[14] Fletcher N.F., Meeker R.B., Hudson L.C., Callanan J.J. (2011). The neuropathogenesis of feline immunodeficiency virus infection: Barriers to overcome. Vet. J..

[15] Hopper C., Sparkes A., Gruffydd-Jones T., Crispin S., Muir P., Harbour D., Stokes C. (1989). Clinical and laboratory findings in cats infected with feline immunodeficiency virus. Vet. Rec..

[16] Lappin M. (1995). Opportunistic infections associated with retroviral infections in cats. Semin. Vet. Med. Surg. Small Anim..

[17] Meeker R.B., Hudson L. (2017). Feline immunodeficiency virus neuropathogenesis: A model for HIV-induced cns inflammation and neurodegeneration. Vet. Sci..

[18] Miller C., Bielefeldt-Ohmann H., MacMillan M., Huitron-Resendiz S., Henriksen S., Elder J., VandeWoude S. (2011). Strain-specific viral distribution and neuropathology of feline immunodeficiency virus. Vet. Immunol. Immunopathol..

[19] Miller C., Boegler K., Carver S., MacMillan M., Bielefeldt-Ohmann H., VandeWoude S. (2017). Pathogenesis of oral fiv infection. PLoS ONE.

[20] Tenorio A.P., Franti C.E., Madewell B.R., Pedersen N.C. (1991). Chronic oral infections of cats and their relationship to persistent oral carriage of feline calici-, immunodeficiency, or leukemia viruses. Vet. Immunol. Immunopathol..

[21] Yamamoto J., Hansen H., Ho E., Morishita T., Okuda T., Sawa T., Nakamura R., Pedersen N. (1989). Epidemiologic and clinical aspects of feline immunodeficiency virus infection in cats from the continental united states and canada and possible mode of transmission. J. Am. Vet. Med. Assoc..

[22] Pedersen N. (1990). Feline immunodeficiency virus infection. Animal Models in AIDS: International TNO Meeting, Maastricht, The Netherlands, 23–26 October 1989.

[23] Del Fierro G., Meers J., Thomas J., Chadwick B., Park H., Robinson W. (1995). Quantification of lymphadenopathy in experimentally induced feline immunodeficiency virus infection in domestic cats. Vet. Immunol. Immunopathol..

[24] Bendinelli M., Pistello M., Lombardi S., Poli A., Garzelli C., Matteucci D., Ceccherini-Nelli L., Malvaldi G., Tozzini F. (1995). Feline immunodeficiency virus: An interesting model for aids studies and an important cat pathogen. Clin. Microbiol. Rev..

[25] Lecollinet S., Richardson J. (2008). Vaccination against the feline immunodeficiency virus: The road not taken. Comp. Immunol. Microbiol. Infect. Dis..

[26] Taniwaki S.A., Figueiredo A.S., Araujo J.P. (2013). Virus–host interaction in feline immunodeficiency virus (FIV) infection. Comp. Immunol. Microbiol. Infect. Dis..

[27] Beatty J.A., Willett B.J., Gault E.A., Jarrett O. (1996). A longitudinal study of feline immunodeficiency virus-specific cytotoxic t lymphocytes in experimentally infected cats, using antigen-specific induction. J. Virol..

[28] Guiot A.-L., Rigal D., Chappuis G. (1997). Spontaneous programmed cell death (pcd) process of lymphocytes of fiv-infected cats: Cellular targets and modulation. Vet. Immunol. Immunopathol..

[29] Hughes M., Ball N., Love D., Canfield P., Wigney D., Dawson D., Davis P., Malik R. (1999). Disseminated mycobacterium genavense infection in a fiv-positive cat. J. Feline Med. Surg..

[30] Poli A., Tozon N., Guidi G., Pistello M. (2012). Renal alterations in feline immunodeficiency virus (FIV)-infected cats: A natural model of lentivirus-induced renal disease changes. Viruses.

[31] Diehl L.J., Mathiason-Dubard C.K., O’Neil L.L., Obert L.A., Hoover E.A. (1995). Induction of accelerated feline immunodeficiency virus disease by acute-phase virus passage. J. Virol..

[32] Kornya M.R., Little S.E., Scherk M.A., Sears W.C., Bienzle D. (2014). Association between oral health status and retrovirus test results in cats. J. Am. Vet. Med. Assoc..

[33] De Rozières S., Mathiason C.K., Rolston M.R., Chatterji U., Hoover E.A., Elder J.H. (2004). Characterization of a highly pathogenic molecular clone of feline immunodeficiency virus clade C. J. Virol..

[34] Weese S.J., Nichols J., Jalali M., Litster A. (2015). The oral and conjunctival microbiotas in cats with and without feline immunodeficiency virus infection. Vet. Res..

[35] Mancianti F., Giannelli C., Bendinelli M., Poli A. (1992). Mycological findings in feline immunodeficiency virus-infected cats. J. Med. Vet. Mycol..

[36] Pennisi M.G. (2015). Leishmaniosis of companion animals in europe: An update. Vet. Parasitol..

[37] Sparkes A., Hopper C., Millard W., Gruffydd-Jones T., Harbour D. (1993). Feline immunodeficiency virus infection clinicopathologic findings in 90 naturally occurring cases. J. Vet. Intern. Med..

[38] Hartmann K. (1998). Feline immunodeficiency virus infection: An overview. Vet. J..

[39] Callanan J., Jones B., Irvine J., Willett B., McCandlish I., Jarrett O. (1996). Histologic classification and immunophenotype of lymphosarcomas in cats with naturally and experimentally acquired feline immunodeficiency virus infections. Vet. Pathol..

[40] English R., Nelson P., Johnson C.M., Nasisse M., Tompkins W.A., Tompkins M.B. (1994). Development of clinical disease in cats experimentally infected with feline immunodeficiency virus. J. Infect. Dis..

[41] Gabor L., Love D., Malik R., Canfield P. (2001). Feline immunodeficiency virus status of australian cats with lymphosarcoma. Aust. Vet. J..

[42] Beatty J. (2014). Viral causes of feline lymphoma: Retroviruses and beyond. Vet. J..

[43] Endo Y., Cho K.-W., Nishigaki K., Momoi Y., Nishimura Y., Mizuno T., Goto Y., Watari T., Tsujimoto H., Hasegawa A. (1997). Molecular characteristics of malignant lymphomas in cats naturally infected with feline immunodeficiency virus. Vet. Immunol. Immunopathol..

[44] Shiramizu B., Herndier B.G., McGrath M.S. (1994). Identification of a common clonal human immunodeficiency virus integration site in human immunodeficiency virus-associated lymphomas. Cancer Res..

[45] Beatty J., Lawrence C., Callanan J., Grant C., Gault E., Neil J., Jarrett O. (1998). Feline immunodeficiency virus (FIV)-associated lymphoma: A potential role for immune dysfunction in tumourigenesis. Vet. Immunol. Immunopathol..

[46] Yamamoto H., Umemura T., Inoshima Y., Nakamura M., Adachi I., Miyazawa T., Mikami T. (1997). Immunological and histological disorders in cats experimentally infected with feline immunodeficiency virus subtype b (TM2 strain). Vet. Microbiol..

[47] Poli A., Abramo F., Taccini E., Guidi G., Barsotti E., Bendinelli M., Malvaldi G. (1993). Renal involvement in feline immunodeficiency virus infection: A clinicopathological study. Nephron.

[48] Matsumoto H., Takemura N., Sako T., Koyama H., Motoyoshi S., Inada Y. (1997). Serum concentration of circulating immune complexes in cats infected with feline immunodeficiency virus detected by immune adherence hemagglutination method. J. Vet. Med. Sci..

[49] Poli A., Falcone M., Bigalli L., Massi C., Hofmann-Lehmann R., Lombardi S., Bendinelli M., Lutz H. (1995). Circulating immune complexes and analysis of renal immune deposits in feline immunodeficiency virus-infected cats. Clin. Exp. Immunol..

[50] Burdo T.H., Miller A.D. (2014). Animal models of HIV peripheral neuropathy. Future Virol..

[51] Podell M., March P.A., Buck W.R., Mathes L.E. (2000). The feline model of neuroaids: Understanding the progression towards aids dementia. J. Psychopharmacol..

[52] Zhu Y., Antony J., Liu S., Martinez J.A., Giuliani F., Zochodne D., Power C. (2006). CD8+ lymphocyte-mediated injury of dorsal root ganglion neurons during lentivirus infection: CD154-dependent cell contact neurotoxicity. J. Neurosci..

[53] Power C., Buist R., Johnston J., Del Bigio M., Ni W., Dawood M., Peeling J. (1998). Neurovirulence in feline immunodeficiency virus-infected neonatal cats is viral strain specific and dependent on systemic immune suppression. J. Virol..

[54] Abramo F., BO S., Canese M.G., Poli A. (1995). Regional distribution of lesions in the central nervous system of cats infected with feline immunodeficiency virus. AIDS Res. Hum. Retrovir..

[55] Steigerwald E.S., Sarter M., March P., Podell M. (1999). Effects of feline immunodeficiency virus on cognition and behavioral function in cats. J. Acquir. Immune Defic. Syndr..

[56] Maingat F., Vivithanaporn P., Zhu Y., Taylor A., Baker G., Pearson K., Power C. (2009). Neurobehavioral performance in feline immunodeficiency virus infection: Integrated analysis of viral burden, neuroinflammation, and neuronal injury in cortex. J. Neurosci..

[57] Phillips T., Prospero-Garcia O., Wheeler D., Wagaman P., Lerner D., Fox H., Whalen L., Bloom F., Elder J., Henriksen S. (1996). Neurologic dysfunctions caused by a molecular clone of feline immunodeficiency virus, FIV-PPR. J. Neurovirol..

[58] Phipps A.J., Hayes K.A., Buck W.R., Podell M., Mathes L.E. (2000). Neurophysiologic and immunologic abnormalities associated with feline immunodeficiency virus molecular clone FIV-PPR DNA inoculation. J. Acquir. Immune Defic. Syndr. 1999.

[59] Sparger E.E. (2006). Fiv as a model for HIV: An overview. In Vivo Models of HIV Disease and Control.

[60] Kenyon J.C., Lever A.M. (2011). The molecular biology of feline immunodeficiency virus (FIV). Viruses.

[61] Steinman R., Dombrowski J., O’Connor T., Montelaro R.C., Tonelli Q., Lawrence K., Seymour C., Goodness J., Pedersen N.C., Andersen P.R. (1990). Biochemical and immunological characterization of the major structural proteins of feline immunodeficiency virus. J Gen. Virol..

[62] Hu Q.-Y., Fink E., Hong Y., Wang C., Grant C.K., Elder J.H. (2010). Fine definition of the cxcr4-binding region on the v3 loop of feline immunodeficiency virus surface glycoprotein. PLoS ONE.

[63] Egberink H.F., Ederveen J., Montelaro R.C., Pedersen N.C., Horzinek M.C., Koolen M.J. (1990). Intracellular proteins of feline immunodeficiency virus and their antigenic relationship with equine infectious anaemia virus proteins. J. Gen. Virol..

[64] Elder J., Schnölzer M., Hasselkus-Light C., Henson M., Lerner D., Phillips T., Wagaman P., Kent S. (1993). Identification of proteolytic processing sites within the gag and pol polyproteins of feline immunodeficiency virus. J. Virol..

[65] Von der Helm K. (1996). Retroviral proteases: Structure, function and inhibition-from a non-anticipated viral enzyme to the target of a most promising HIV therapy. Biol. Chem. Hoppe Seyler.

[66] Gadsden M.H., McIntosh E., Game J.C., Wilson P.J., Haynes R. (1993). Dutp pyrophosphatase is an essential enzyme in saccharomyces cerevisiae. EMBO J..

[67] Khan E., Mack J.P., Katz R.A., Kulkosky J., Skalka A.M. (1991). Retroviral integrase domains: DNA binding and the recognition of ltr sequences. Nucleic Acids Res..

[68] Vink C., van der Linden K.H., Plasterk R. (1994). Activities of the feline immunodeficiency virus integrase protein produced in escherichia coli. J. Virol..

[69] North T.W., Cronn R.C., Remington K.M., Tandberg R.T., Judd R.C. (1990). Characterization of reverse transcriptase from feline immunodeficiency virus. J. Biol. Chem..

[70] Foley B.T., Leitner T.K., Apetrei C., Hahn B., Mizrachi I., Mullins J., Rambaut A., Wolinsky S., Korber B.T.M. (2015). HIV Sequence Compendium 2015.

[71] De Parseval A., Elder J.H. (2001). Binding of recombinant feline immunodeficiency virus surface glycoprotein to feline cells: Role of cxcr4, cell-surface heparans, and an unidentified non-cxcr4 receptor. J. Virol..

[72] De Parseval A., Ngo S., Sun P., Elder J.H. (2004). Factors that increase the effective concentration of cxcr4 dictate feline immunodeficiency virus tropism and kinetics of replication. J. Virol..

[73] De Parseval A., Chatterji U., Sun P., Elder J.H. (2004). Feline immunodeficiency virus targets activated cd4+ t cells by using CD134 as a binding receptor. Proc. Natl. Acad. Sci. USA.

[74] De Parseval A., Grant C.K., Sastry K.J., Elder J.H. (2006). Sequential CD134-CXCR4 interactions in feline immunodeficiency virus (FIV): Soluble CD134 activates FIV Env for CXCR4-dependent entry and reveals a cryptic neutralization epitope. J. Virol..

[75] Sundstrom M., White R.L., de Parseval A., Sastry K.J., Morris G., Grant C.K., Elder J.H. (2008). Mapping of the cxcr4 binding site within variable region 3 of the feline immunodeficiency virus surface glycoprotein. J. Virol..

[76] Garg H., Fuller F.J., Tompkins W.A. (2004). Mechanism of feline immunodeficiency virus envelope glycoprotein-mediated fusion. Virology.

[77] Dean G.A., Reubel G.H., Moore P.F., Pedersen N.C. (1996). Proviral burden and infection kinetics of feline immunodeficiency virus in lymphocyte subsets of blood and lymph node. J. Virol..

[78] Willett B.J., Hosie M.J. (2008). Chemokine receptors and co-stimulatory molecules: Unravelling feline immunodeficiency virus infection. Vet. Immunol. Immunopathol..

[79] Hosie M.J., Pajek D., Samman A., Willett B.J. (2011). Feline immunodeficiency virus (FIV) neutralization: A review. Viruses.

[80] Willett B.J., Hosie M.J. (2013). The virus–receptor interaction in the replication of feline immunodeficiency virus (FIV). Curr. Opin. in Virol..

[81] Berger E.A., Murphy P.M., Farber J.M. (1999). Chemokine receptors as HIV-1 coreceptors: Roles in viral entry, tropism, and disease. Ann. Rev. Immunol..

[82] Doms R.W. (2001). Chemokine receptors and HIV entry. AIDS.

[83] Elder J.H., Sundstrom M., de Rozieres S., de Parseval A., Grant C.K., Lin Y.-C. (2008). Molecular mechanisms of fiv infection. Vet. Immunol. Immunopathol..

[84] Mosier D.E. (2009). How HIV changes its tropism: Evolution and adaptation?. Curr. Opin. HIV AIDS.

[85] Peters P.J., Duenas-Decamp M.J., Sullivan W.M., Brown R., Ankghuambom C., Luzuriaga K., Robinson J., Burton D.R., Bell J., Simmonds P. (2008). Variation in HIV-1 R5 macrophage-tropism correlates with sensitivity to reagents that block envelope: CD4 interactions but not with sensitivity to other entry inhibitors. Retrovirology.

[86] Clapham P.R., McKnight A. (2001). HIV-1 receptors and cell tropism. Br. Med. Bull..

[87] Regoes R.R., Bonhoeffer S. (2005). The HIV coreceptor switch: A population dynamical perspective. Trends Microbiol..

[88] Tomonaga K., Mikami T. (1996). Molecular biology of the feline immunodeficiency virus auxiliary genes. J. Gen. Virol..

[89] LaRue R.S., Lengyel J., Jónsson S.R., Andrésdóttir V., Harris R.S. (2010). Lentiviral vif degrades the apobec3z3/apobec3h protein of its mammalian host and is capable of cross-species activity. J. Virol..

[90] Sundstrom M., Chatterji U., Schaffer L., de Rozières S., Elder J.H. (2008). Feline immunodeficiency virus orfa alters gene expression of splicing factors and proteasome-ubiquitination proteins. Virology.

[91] Gemeniano M.C., Sawai E.T., Leutenegger C.M., Sparger E.E. (2003). Feline immunodeficiency virus orf-a is required for virus particle formation and virus infectivity. J. Virol..

[92] Waters A., De Parseval A., Lerner D., Neil J., Thompson F., Elder J. (1996). Influence of ORF2 on host cell tropism of feline immunodeficiency virus. Virology.

[93] De Parseval A., Elder J.H. (1999). Demonstration that Orf2 encodes the feline immunodeficiency virus transactivating (TAT) protein and characterization of a unique gene product with partial rev activity. J. Virol..

[94] Gemeniano M.C., Sawai E.T., Sparger E.E. (2004). Feline immunodeficiency virus Orf-A localizes to the nucleus and induces cell cycle arrest. Virology.

[95] Hong Y., Fink E., Hu Q.-Y., Kiosses W.B., Elder J.H. (2010). Orfa downregulates feline immunodeficiency virus primary receptor cd134 on the host cell surface and is important in viral infection. J. Virol..

[96] Miller R.H. (1988). Human immunodeficiency virus may encode a novel protein on the genomic DNA plus strand. Science.

[97] Briquet S., Vaquero C. (2002). Immunolocalization studies of an antisense protein in HIV-1-infected cells and viral particles. Virology.

[98] Bukrinsky M.I., Etkin A.F. (1990). Plus strand of the HIV provirus DNA is expressed at early stages of infection. AIDS Res. Hum. Retrovir..

[99] Kobayashi-Ishihara M., Yamagishi M., Hara T., Matsuda Y., Takahashi R., Miyake A., Nakano K., Yamochi T., Ishida T., Watanabe T. (2012). HIV-1-encoded antisense rna suppresses viral replication for a prolonged period. Retrovirology.

[100] Landry S., Halin M., Lefort S., Audet B., Vaquero C., Mesnard J.M., Barbeau B. (2007). Detection, characterization and regulation of antisense transcripts in HIV-1. Retrovirology.

[101] Laverdure S., Gross A., Arpin-Andre C., Clerc I., Beaumelle B., Barbeau B., Mesnard J.M. (2012). HIV-1 antisense transcription is preferentially activated in primary monocyte-derived cells. J. Virol..

[102] Ludwig L.B., Ambrus J.L., Krawczyk K.A., Sharma S., Brooks S., Hsiao C.B., Schwartz S.A. (2006). Human immunodeficiency virus-type 1 LTR DNA contains an intrinsic gene producing antisense RNA and protein products. Retrovirology.

[103] Michael N.L., Vahey M.T., d’Arcy L., Ehrenberg P.K., Mosca J.D., Rappaport J., Redfield R.R. (1994). Negative-strand rna transcripts are produced in human immunodeficiency virus type 1-infected cells and patients by a novel promoter downregulated by tat. J. Virol..

[104] Torresilla C., Do Carmo S., Larocque É., Douceron E., Mesnard J.-M., Mahieux R., Barbeau B. (2014). The antisense protein of HTLV-2 positively modulates HIV-1 replication. Retrovirology.

[105] Torresilla C., Larocque E., Landry S., Halin M., Coulombe Y., Masson J.Y., Mesnard J.M., Barbeau B. (2013). Detection of the HIV-1 minus-strand-encoded antisense protein and its association with autophagy. J. Virol..

[106] Vanhee-Brossollet C., Thoreau H., Serpente N., D’Auriol L., Levy J.P., Vaquero C. (1995). A natural antisense rna derived from the HIV-1 env gene encodes a protein which is recognized by circulating antibodies of HIV+ individuals. Virology.

[107] Cassan E., Arigon-Chifolleau A.M., Mesnard J.M., Gross A., Gascuel O. (2016). Concomitant emergence of the antisense protein gene of HIV-1 and of the pandemic. Proc. Natl. Acad. Sci. USA.

[108] Briquet S., Richardson J., Vanhee-Brossollet C., Vaquero C. (2001). Natural antisense transcripts are detected in different cell lines and tissues of cats infected with feline immunodeficiency virus. Gene.

[109] Durkin K., Rosewick N., Artesi M., Hahaut V., Griebel P., Arsic N., Burny A., Georges M., Van den Broeke A. (2016). Characterization of novel bovine leukemia virus (BLV) antisense transcripts by deep sequencing reveals constitutive expression in tumors and transcriptional interaction with viral micrornas. Retrovirology.

[110] McCune J.M. (2001). The dynamics of CD4+ t-cell depletion in HIV disease. Nature.

[111] Obert L.A., Hoover E.A. (2002). Early pathogenesis of transmucosal feline immunodeficiency virus infection. J. Virol..

[112] Obert L., Hoover E. (2000). Relationship of lymphoid lesions to disease course in mucosal feline immunodeficiency virus type c infection. Vet. Pathol..

[113] Woo J.C., Dean G.A., Pedersen N.C., Moore P.F. (1997). Immunopathologic changes in the thymus during the acute stage of experimentally induced feline immunodeficiency virus infection in juvenile cats. J. Virol..

[114] Serrano-Villar S., Sainz T., Lee S.A., Hunt P.W., Sinclair E., Shacklett B.L., Ferre A.L., Hayes T.L., Somsouk M., Hsue P.Y. (2014). HIV-infected individuals with low CD4/CD8 ratio despite effective antiretroviral therapy exhibit altered T cell subsets, heightened CD8+ T cell activation, and increased risk of non-aids morbidity and mortality. PLoS Pathog..

[115] Loughran T.J. (1993). Clonal diseases of large granular lymphocytes [see comments]. Blood.

[116] Phillips J., Lanier L. (1986). Lectin-dependent and anti-CD3 induced cytotoxicity are preferentially mediated by peripheral blood cytotoxic T lymphocytes expressing Leu-7 antigen. J. Immunol..

[117] Schmidt R.E., Murray C., Daley J.F., Schlossman S., Ritz J. (1986). A subset of natural killer cells in peripheral blood displays a mature T cell phenotype. J. Exp. Med..

[118] Sprague W.S., Apetrei C., Avery A.C., Peskind R.L., Vandewoude S. (2015). Large granular lymphocytes are universally increased in human, macaque, and feline lentiviral infection. Vet. Immunol. Immunopathol..

[119] Alekshun T.J., Sokol L. (2007). Diseases of large granular lymphocytes. Cancer Control.

[120] Boveri E., Riboni R., Antico P., Malacrida A., Pastorini A. (2009). CD3+ T large granular lymphocyte leukaemia in a HIV+, HCV+, HBV+ patient. Virchows Arch..

[121] Smith P.R., Cavenagh J.D., Milne T., Howe D., Wilkes S.J., Sinnott P., Forster G.E., Helbert M. (2000). Benign monoclonal expansion of cd8+ lymphocytes in HIV infection. J. Clin. Pathol..

[122] Sprague W., TerWee J., VandeWoude S. (2010). Temporal association of large granular lymphocytosis, neutropenia, proviral load, and fasl mrna in cats with acute feline immunodeficiency virus infection. Vet. Immunol. Immunopathol..

[123] Fogle J.E., Mexas A.M., Tompkins W.A., Tompkins M.B. (2010). CD4+ CD25+ T regulatory cells inhibit CD8+ IFN-γ production during acute and chronic fiv infection utilizing a membrane TGF-β-dependent mechanism. AIDS Res. Hum. Retrovir..

[124] Kinter A.L., Horak R., Sion M., Riggin L., McNally J., Lin Y., Jackson R., O’Shea A., Roby G., Kovacs C. (2007). CD25+ regulatory T cells isolated from HIV-infected individuals suppress the cytolytic and nonlytic antiviral activity of HIV-specific CD8+ t cells in vitro. AIDS Res. Hum. Retrovir..

[125] Miller M.M., Fogle J.E., Tompkins M.B. (2013). Infection with feline immunodeficiency virus directly activates CD4+ CD25+ T regulatory cells. J. Virol..

[126] Petito C.K. (2004). Human immunodeficiency virus type 1 compartmentalization in the central nervous system. J. Neurovirol..

[127] Zenger E., Tiffany-Castiglioni E., Collisson E.W. (1997). Cellular mechanisms of feline immunodeficiency virus (FIV)-induced neuropathogenesis. Front. Biosci..

[128] Fletcher N., Bexiga M., Brayden D., Brankin B., Willett B., Hosie M., Jacque J.M., Callanan J. (2009). Lymphocyte migration through the blood–brain barrier (BBB) in feline immunodeficiency virus infection is significantly influenced by the pre-existence of virus and tumour necrosis factor (TNF)-α within the central nervous system (CNS): Studies using an in vitro feline bbb model. Neuropathol. Appl. Neurobiol..

[129] Hudson L., Bragg D., Tompkins M., Meeker R. (2005). Astrocytes and microglia differentially regulate trafficking of lymphocyte subsets across brain endothelial cells. Brain Res..

[130] González-Scarano F., Martín-García J. (2005). The neuropathogenesis of aids. Nat. Rev. Immunol..

[131] Brinkmann R., Schwinn A., Narayan O., Zink C., Kreth H., Roggendorf W., Dörries R., Schwender S., Imrich H., Ter Meulen V. (1992). Human immunodeficiency virus infection in microglia: Correlation between cells infected in the brain and cells cultured from infectious brain tissue. Ann. Neurol..

[132] Kawaguchi Y., Maeda K., Tohya Y., Furuya T., Miyazawa T., Horimoto T., Norimine J., Kai C., Mikami T. (1992). Replicative difference in early-passage feline brain cells among feline immunodeficiency virus isolates. Arch. Virol..

[133] Poli A., Abramo F., Iorio C.D., Cantile C., Carli M.A., Pollera C., Vago L., Tosoni A., Costanzi G. (1997). Neuropathology in cats experimentally infected wit feline immunodeficiency virus: A morphological, immunocytochemical and morphometric study. J. Neurovirol..

[134] Meeker R.B., Poulton W., Feng W.-H., Hudson L., Longo F.M. (2012). Suppression of immunodeficiency virus-associated neural damage by the P75 neurotrophin receptor ligand, LM11A-31, in an in vitro feline model. J. Neuroimmune Pharmacol..

[135] Liu P., Hudson L.C., Tompkins M.B., Vahlenkamp T.W., Meeker R.B. (2006). Compartmentalization and evolution of feline immunodeficiency virus between the central nervous system and periphery following intracerebroventricular or systemic inoculation. J. Neurovirol..

[136] Kennedy J.M., Hoke A., Zhu Y., Johnston J.B., van Marle G., Silva C., Zochodne D.W., Power C. (2004). Peripheral neuropathy in lentivirus infection: Evidence of inflammation and axonal injury. Aids.

[137] Burdo T.H., Lackner A., Williams K.C. (2013). Monocyte/macrophages and their role in HIV neuropathogenesis. Immunol. Rev..

[138] Williams K., Burdo T.H. (2012). Monocyte mobilization, activation markers, and unique macrophage populations in the brain: Observations from siv infected monkeys are informative with regard to pathogenic mechanisms of HIV infection in humans. J. Neuroimmune Pharmacol..

[139] Williams K., Lackner A., Mallard J. (2016). Non-human primate models of siv infection and cns neuropathology. Curr. Opin. Virol..

[140] Williams K.C., Hickey W.F. (2002). Central nervous system damage, monocytes and macrophages, and neurological disorders in aids. Ann. Rev. Neurosci..

[141] Laast V.A., Pardo C.A., Tarwater P.M., Queen S.E., Reinhart T.A., Ghosh M., Adams R.J., Zink M.C., Mankowski J.L. (2007). Pathogenesis of simian immunodeficiency virus-induced alterations in macaque trigeminal ganglia. J. Neuropathol. Exp. Neurol..

[142] Laast V.A., Shim B., Johanek L.M., Dorsey J.L., Hauer P.E., Tarwater P.M., Adams R.J., Pardo C.A., McArthur J.C., Ringkamp M. (2011). Macrophage-mediated dorsal root ganglion damage precedes altered nerve conduction in siv-infected macaques. Am. J. Pathol..

[143] Lehmann H.C., Chen W., Borzan J., Mankowski J.L., Höke A. (2011). Mitochondrial dysfunction in distal axons contributes to human immunodeficiency virus sensory neuropathy. Ann Neurol..

[144] VandeWoude S., Apetrei C. (2006). Going wild: Lessons from naturally occurring T-lymphotropic lentiviruses. Clin. Microbiol. Rev..

[145] Santos R.V., Lin K.-C., Mansfield K., Wachtman L.M. (2011). Specific pathogen-free status alters immunophenotype in rhesus macaques: Implications for the study of simian immunodeficiency virus. AIDS Res. Hum. Retrovir..

[146] Denton P.W., Garcia J.V. (2011). Humanized mouse models of HIV infection. AIDS Rev..

[147] Zheng J., Ghorpade A., Niemann D., Cotter R.L., Thylin M.R., Epstein L., Swartz J.M., Shepard R.B., Liu X., Nukuna A. (1999). Lymphotropic virions affect chemokine receptor-mediated neural signaling and apoptosis: Implications for human immunodeficiency virus type 1-associated dementia. J. Virol..

[148] Hesselgesser J., Taub D., Baskar P., Greenberg M., Hoxie J., Kolson D.L., Horuk R. (1998). Neuronal apoptosis induced by HIV-1 GP120 and the chemokine SDF-1 alpha is mediated by the chemokine receptor CXCR4. Curr. Biol. CB.

[149] Zheng J., Thylin M.R., Ghorpade A., Xiong H., Persidsky Y., Cotter R., Niemann D., Che M., Zeng Y.C., Gelbard H.A. (1999). Intracellular CXCR4 signaling, neuronal apoptosis and neuropathogenic mechanisms of HIV-1-associated dementia. J. Neuroimmunol..

[150] Meucci O., Fatatis A., Simen A.A., Bushell T.J., Gray P.W., Miller R.J. (1998). Chemokines regulate hippocampal neuronal signaling and GP120 neurotoxicity. Proc. Natl. Acad. Sci. USA.

[151] Lipton S.A., Sucher N.J., Kaiser P.K., Dreyer E.B. (1991). Synergistic effects of HIV coat protein and nmda receptor-mediated neurotoxicity. Neuron.

[152] Haughey N.J., Mattson M.P. (2002). Calcium dysregulation and neuronal apoptosis by the HIV-1 proteins tat and GP120. J. Acquir. Immune Defic. Syndr..

[153] Ballester L.Y., Capo-Velez C.M., Garcia-Beltran W.F., Ramos F.M., Vazquez-Rosa E., Rios R., Mercado J.R., Melendez R.I., Lasalde-Dominicci J.A. (2012). Up-regulation of the neuronal nicotinic receptor alpha7 by HIV glycoprotein 120: Potential implications for HIV-associated neurocognitive disorder. J. Biol. Chem..

[154] Bredt D.S., Snyder S.H. (1989). Nitric oxide mediates glutamate-linked enhancement of CGMP levels in the cerebellum. Proc. Natl. Acad. Sci. USA.

[155] Garthwaite J., Garthwaite G., Palmer R.M., Moncada S. (1989). Nmda receptor activation induces nitric oxide synthesis from arginine in rat brain slices. Eur. J. Pharmacol..

[156] Kornau H.C., Seeburg P.H., Kennedy M.B. (1997). Interaction of ion channels and receptors with PDZ domain proteins. Curr. Opin. Neurobiol..

[157] Christopherson K.S., Hillier B.J., Lim W.A., Bredt D.S. (1999). PSD-95 assembles a ternary complex with the *N*-methyl-d-aspartic acid receptor and a bivalent neuronal NO synthase PDZ domain. J. Biol. Chem..

[158] Rameau G.A., Tukey D.S., Garcin-Hosfield E.D., Titcombe R.F., Misra C., Khatri L., Getzoff E.D., Ziff E.B. (2007). Biphasic coupling of neuronal nitric oxide synthase phosphorylation to the nmda receptor regulates ampa receptor trafficking and neuronal cell death. J. Neurosci..

[159] Bredt D.S. (2003). Nitric oxide signaling specificity—The heart of the problem. J. Cell Sci..

[160] Bragg D.C., Meeker R.B., Duff B.A., English R.V., Tompkins M.B. (1999). Neurotoxicity of FIV and FIV envelope protein in feline cortical cultures. Brain Res..

[161] Brenneman D.E., Westbrook G.L., Fitzgerald S.P., Ennist D.L., Elkins K.L., Ruff M.R., Pert C.B. (1988). Neuronal cell killing by the envelope protein of HIV and its prevention by vasoactive intestinal peptide. Nature.

[162] Yamamoto J.K., Pu R., Sato E., Hohdatsu T. (2007). Feline immunodeficiency virus pathogenesis and development of a dual-subtype feline-immunodeficiency-virus vaccine. AIDS.

[163] Uhl E., Heaton-Jones T., Pu R., Yamamoto J. (2002). Fiv vaccine development and its importance to veterinary and human medicine: A review: FIV vaccine 2002 update and review. Vet. Immunol. Immunopathol..

[164] Pu R., Coleman J., Coisman J., Sato E., Tanabe T., Arai M., Yamamoto J.K. (2005). Dual-subtype FIV vaccine (Fel-O-Vax^®^ FIV) protection against a heterologous subtype B FIV isolate. J. Feline Med. Surg..

[165] Bęczkowski P.M., Harris M., Techakriengkrai N., Beatty J.A., Willett B.J., Hosie M.J. (2015). Neutralising antibody response in domestic cats immunised with a commercial feline immunodeficiency virus (FIV) vaccine. Vaccine.

[166] Westman M., Malik R., Hall E., Harris M., Norris J. (2016). The protective rate of the feline immunodeficiency virus vaccine: An australian field study. Vaccine.

[167] Dunham S.P., Bruce J., Klein D., Flynn J.N., Golder M.C., MacDonald S., Jarrett O., Neil J.C. (2006). Prime-boost vaccination using DNA and whole inactivated virus vaccines provides limited protection against virulent feline immunodeficiency virus. Vaccine.

[168] Dunham S., Bruce J., MacKay S., Golder M., Jarrett O., Neil J. (2006). Limited efficacy of an inactivated feline immunodeficiency virus vaccine. Vet. Rec..

[169] Hosie M.J., Osborne R., Reid G., Neil J.C., Jarrett O. (1992). Enhancement after feline immunodeficiency virus vaccination. Vet. Immunol. Immunopathol..

[170] Lombardi S., Garzelli C., Pistello M., Massi C., Matteucci D., Baldinotti F., Cammarota G., Da Prato L., Bandecchi P., Tozzini F. (1994). A neutralizing antibody-inducing peptide of the V3 domain of feline immunodeficiency virus envelope glycoprotein does not induce protective immunity. J. Virol..

[171] Siebelink K., Tijhaar E., Huisman R.C., Huisman W., De Ronde A., Darby I.H., Francis M.J., Rimmelzwaan G.F., Osterhaus A. (1995). Enhancement of feline immunodeficiency virus infection after immunization with envelope glycoprotein subunit vaccines. J. Virol..

[172] Richardson J., Moraillon A., Baud S., Cuisinier A., Sonigo P., Pancino G. (1997). Enhancement of feline immunodeficiency virus (FIV) infection after DNA vaccination with the fiv envelope. J. Virol..

[173] Karlas J.A., Siebelink K., Peer M.A.V., Huisman W., Cuisinier A.M., Rimmelzwaan G.F., Osterhaus A. (1999). Vaccination with experimental feline immunodeficiency virus vaccines, based on autologous infected cells, elicits enhancement of homologous challenge infection. J Gen. Virol..

[174] Giannecchini S., Isola P., Sichi O., Matteucci D., Pistello M., Zaccaro L., Del Mauro D., Bendinelli M. (2002). Aids vaccination studies using an ex vivo feline immunodeficiency virus model: Failure to protect and possible enhancement of challenge infection by four cell-based vaccines prepared with autologous lymphoblasts. J. Virol..

[175] Lun W.-H., Takeda A., Nakamura H., Kano M., Mori K., Sata T., Nagai Y., Matano T. (2004). Loss of virus-specific CD4+ T cells with increases in viral loads in the chronic phase after vaccine-based partial control of primary simian immunodeficiency virus replication in macaques. J. Gen. Virol..

[176] Mueller Y.M., Do D.H., Altork S.R., Artlett C.M., Gracely E.J., Katsetos C.D., Legido A., Villinger F., Altman J.D., Brown C.R. (2008). Il-15 treatment during acute simian immunodeficiency virus (SIV) infection increases viral set point and accelerates disease progression despite the induction of stronger siv-specific CD8+ t cell responses. J. Immunol..

[177] Robinson W.E., Montefiori D., Mitchell W. (1988). Antibody-dependent enhancement of human immunodeficiency virus type 1 infection. Lancet.

[178] Staprans S.I., Hamilton B.L., Follansbee S.E., Elbeik T., Barbosa P., Grant R.M., Feinberg M.B. (1995). Activation of virus replication after vaccination of HIV-1-infected individuals. J. Exp. Med..

[179] Villinger F., Rowe T., Parekh B.S., Green T.A., Mayne A.E., Grimm B., McClure H.M., Lackner A.A., Dailey P.J., Ansari A.A. (2001). Chronic immune stimulation accelerates siv-induced disease progression. J. Med. Primatol..

[180] Staprans S.I., Barry A.P., Silvestri G., Safrit J.T., Kozyr N., Sumpter B., Nguyen H., McClure H., Montefiori D., Cohen J.I. (2004). Enhanced siv replication and accelerated progression to aids in macaques primed to mount a cd4 t cell response to the siv envelope protein. Proc. Natl. Acad. Sci. USA.

[181] Huisman W., Martina B., Rimmelzwaan G., Gruters R., Osterhaus A. (2009). Vaccine-induced enhancement of viral infections. Vaccine.

[182] Montefiori D.C. (1997). Role of complement and FC receptors in the pathogenesis of HIV-1 infection. Immunopathogenesis of HIV Infection.

[183] Müller-Eberhard H.J. (1988). Molecular organization and function of the complement system. Ann. Rev. Biochem..

[184] Willey S., Aasa-Chapman M.M., O’Farrell S., Pellegrino P., Williams I., Weiss R.A., Neil S.J. (2011). Extensive complement-dependent enhancement of HIV-1 by autologous non-neutralising antibodies at early stages of infection. Retrovirology.

[185] Szabó J., Prohászka Z., Tóth F.D., Gyuris Á., Segesdi J., Bánhegyi D., Ujhelyi E., Minárovits J., Füst G. (1999). Strong correlation between the complement-mediated antibody-dependent enhancement of HIV-1 infection and plasma viral load. AIDS.

[186] Robinson W.E., Kawamura T., Lake D., Masuho Y., Mitchell W., Hersh E.M. (1990). Antibodies to the primary immunodominant domain of human immunodeficiency virus type 1 (HIV-1) glycoprotein gp41 enhance HIV-1 infection in vitro. J. Virol..

[187] Robinson W., Gorny M., Xu J., Mitchell W., Zolla-Pazner S. (1991). Two immunodominant domains of GP41 bind antibodies which enhance human immunodeficiency virus type 1 infection in vitro. J. Virol..

[188] Montefiori D.C., Robinson W.E., Mitchell W.M. (1989). Antibody-independent, complement-mediated enhancement of HIV-1 infection by mannosidase i and ii inhibitors. Antivir. Res..

[189] Boyer V., Desgranges C., Trabaud M., Fischer E., Kazatchkine M. (1991). Complement mediates human immunodeficiency virus type 1 infection of a human t cell line in a CD4- and antibody-independent fashion. J. Exp. Med..

[190] Montefiori D.C., Stewart K., Ahearn J.M., Zhou J. (1993). Complement-mediated binding of naturally glycosylated and glycosylation-modified human immunodeficiency virus type 1 to human CR2 (CD21). J. Virol..

[191] Reisinger E.C., Vogetseder W., Berzow D., Köfler D., Bitterlich G., Lehr H.A., Wachter H., Dierich M.P. (1990). Complement-mediated enhancement of HIV-1 infection of the monoblastoid cell line U937. AIDS.

[192] Sölder B., Schulz T., Hengster P., Löwer J., Larcher C., Bitterlich G., Kurth R., Wachter H., Dierich M. (1989). HIV and HIV-infected cells differentially activate the human complement system independent of antibody. Immunol. Lett..

[193] Spear G.T., Jiang H., Sullivan B.L., Gewürz H., Landay A.L., Lint T.F. (1991). Direct binding of complement component C1q to human immunodeficiency virus (HIV) and human T lymphotrophic virus-I (HTLV-I) coinfected cells. AIDS Res. Hum. Retrovir..

[194] Richardson J., Broche S., Baud S., Leste-Lasserre T., Féménia F., Levy D., Moraillon A., Pancino G., Sonigo P. (2002). Lymphoid activation: A confounding factor in aids vaccine development?. J. Gen. Virol..

[195] Wahl S.M., Greenwell-Wild T., Peng G., Hale-Donze H., Orenstein J.M. (1999). Co-infection with opportunistic pathogens promotes human immunodeficiency virus type 1 infection in macrophages. J. Infect. Dis..

[196] Wahl S., Orenstein J.M. (1997). Immune stimulation and HIV-1 viral replication. J. Leukoc. Biol..

[197] Wu S.-C., Spouge J.L., Conley S.R., Tsai W.-P., Merges M.J., Nara P.L. (1995). Human plasma enhances the infectivity of primary human immunodeficiency virus type 1 isolates in peripheral blood mononuclear cells and monocyte-derived macrophages. J. Virol..

[198] Thibault S., Tardif M.R., Barat C., Tremblay M.J. (2007). TLR2 signaling renders quiescent naive and memory CD4+ T cells more susceptible to productive infection with X4 and R5 HIV-type 1. J. Immunol..

[199] Daniel M.D., Kirchhoff F., Czajak S.C., Sehgal P.K., Desrosiers R.C. (1992). Protective effects of a live attenuated siv vaccine with a deletion in the nef gene. Science.

[200] Evans D.T., Silvestri G. (2013). Non-human primate models in aids research. Curr. Opin. HIV AIDS.

[201] Hessell A.J., Hangartner L., Hunter M., Havenith C.E., Beurskens F.J., Bakker J.M., Lanigan C.M., Landucci G., Forthal D.N., Parren P.W. (2007). Fc receptor but not complement binding is important in antibody protection against HIV. Nature.

[202] Mascola J.R., Stiegler G., VanCott T.C., Katinger H., Carpenter C.B., Hanson C.E., Beary H., Hayes D., Frankel S.S., Birx D.L. (2000). Protection of macaques against vaginal transmission of a pathogenic HIV-1/SIV chimeric virus by passive infusion of neutralizing antibodies. Nat. Med..

[203] Hatziioannou T., Evans D.T. (2012). Animal models for HIV/AIDs research. Nat. Rev. Microbiol..

[204] Gauduin M.-C., Parren P.W., Weir R., Barbas C.F., Burton D.R., Koup R.A. (1997). Passive immunization with a human monoclonal antibody protects hu-PBL-SCID mice against challenge by primary isolates of HIV-1. Nat. Med..

[205] Jenkins M., Hanley M.B., Moreno M.B., Wieder E., McCune J.M. (1998). Human immunodeficiency virus-1 infection interrupts thymopoiesis and multilineage hematopoiesis in vivo. Blood.

[206] McCune J.M., Namikawa R., Shih C.-C., Rabin L., Kaneshima H. (1990). Suppression of HIV infection in AZT-treated SCID-HU mice. Science.

[207] Safrit J.T., Fung M.S., Andrews C.A., Braun D.G., Sun W.N., Chang T.W., Koup R.A. (1993). Hu-PBL-SCID mice can be protected from HIV-1 infection by passive transfer of monoclonal antibody to the principal neutralizing determinant of envelope GP120. AIDS.

[208] Mataftsi M., Skoura L., Sakellari D. (2011). Hiv infection and periodontal diseases: An overview of the post-HAART era. Oral Dis..

[209] Armitage G.C. (1999). Development of a classification system for periodontal diseases and conditions. Ann. Periodontol..

[210] Coogan M.M., Greenspan J., Challacombe S.J. (2005). Oral lesions in infection with human immunodeficiency virus. Bull. World Health Organ..

[211] Miziara I.D., Weber R. (2008). Oral lesions as predictors of highly active antiretroviral therapy failure in brazilian HIV-infected children. J. Oral Pathol. Med..

[212] Soares L.F., de Araújo Castro G.F.B., de Souza I.P.R., Pinheiro M. (2004). Pediatric HIV-related oral manifestations: A five-year retrospective study. Braz. Oral Res..

[213] Vaseliu N., Carter A.B., Kline N.E., Kozinetz C., Cron S.G., Matusa R., Kline M.W. (2005). Longitudinal study of the prevalence and prognostic implications of oral manifestations in romanian children infected with human immunodeficiency virus type 1. Pediatr. Infect. Dis. J..

[214] Brenchley J.M., Price D.A., Schacker T.W., Asher T.E., Silvestri G., Rao S., Kazzaz Z., Bornstein E., Lambotte O., Altmann D. (2006). Microbial translocation is a cause of systemic immune activation in chronic HIV infection. Nat. Med..

[215] Klatt N.R., Chomont N., Douek D.C., Deeks S.G. (2013). Immune activation and HIV persistence: Implications for curative approaches to HIV infection. Immunol. Rev..

[216] Klatt N.R., Funderburg N.T., Brenchley J.M. (2013). Microbial translocation, immune activation, and HIV disease. Trends Microbiol..

[217] Mehandru S., Poles M.A., Tenner-Racz K., Horowitz A., Hurley A., Hogan C., Boden D., Racz P., Markowitz M. (2004). Primary HIV-1 infection is associated with preferential depletion of CD4+ T lymphocytes from effector sites in the gastrointestinal tract. J. Exp. Med..

[218] Brenchley J.M., Price D.A., Douek D.C. (2006). Hiv disease: Fallout from a mucosal catastrophe?. Nat. Immunol..

[219] Baskin G., Murphey-Corb M., Watson E., Martin L. (1988). Necropsy findings in rhesus monkeys experimentally infected with cultured simian immunodeficiency virus (SIV)/delta. Vet. Pathol. Online.

[220] Casteleyn C., Breugelmans S., Simoens P., Van den Broeck W. (2011). The tonsils revisited: Review of the anatomical localization and histological characteristics of the tonsils of domestic and laboratory animals. Clin. Dev. Immunol..

[221] McClure H., Anderson D., Fultz P., Ansari A., Lockwood E., Brodie A. (1989). Spectrum of disease in macaque monkeys chronically infected with SIV/SMM. Vet. Immunol. Immunopathol..

[222] Dos Santos L.D.C., Castro G.F., de Souza I.P.R., Oliveira R.H.S. (2001). Oral manifestations related to immunosuppression degree in HIV-positive children. Braz. Dent. J..

[223] Sparkes A., Caney S.M. (2004). Feline Medicine: Self-Assessment Color Review.

[224] Willett B., Flynn N., Hosic M. (1997). FIV infection of the domestic cat: An animal model for aids. Immunol. Today.

[225] Yamamoto J.K., Sparger E., Ho E.W., Andersen P.R., O’connor T., Mandell C., Lowenstine L., Munn R., Pedersen N. (1988). Pathogenesis of experimentally induced feline immunodeficiency virus infection in cats. Am. J. Vet. Res..

[226] Brady L., Walker C., Oxford G., Stewart C., Magnusson I., McArthur W. (1996). Oral diseases, mycology and periodontal microbiology of HIV-1-infected women. Mol. Oral Microbiol..

[227] Flaitz C.M., Hicks M.J. (1999). Oral candidiasis in children with immune suppression: Clinical appearance and therapeutic considerations. ASDC J. Dent. Child..

[228] Holt S.C., Ebersole J.L. (2005). Porphyromonas gingivalis, treponema denticola, and tannerella forsythia: The ‘red complex’, a prototype polybacterial pathogenic consortium in periodontitis. Periodontology 2000.

[229] Li Y., Saxena D., Chen Z., Liu G., Abrams W.R., Phelan J.A., Norman R.G., Fisch G.S., Corby P.M., Dewhirst F. (2014). HIV infection and microbial diversity in saliva. J. Clin. Microbiol..

[230] Love D., Johnson J., Moore L. (1989). Bacteroides species from the oral cavity and oral-associated diseases of cats. Vet. Microbiol..

[231] Murray P.A., Grassi M., Winkler J.R. (1989). The microbiology of HIV-associated periodontal lesions. J. Clin. Periodontol..

[232] Nokta M. (2008). Oral manifestations associated with HIV infection. Curr. HIV/AIDS Rep..

[233] Norris J.M., Love D.N. (1999). Associations amongst three feline porphyromonas species from the gingival margin of cats during periodontal health and disease. Vet. Microbiol..

[234] Rams T.E., Andriolo M., Feik D., Abel S.N., McGivern T.M., Slots J. (1991). Microbiological study of HIV-related periodontitis. J. Periodontol..

[235] Sims T., Moncla B., Page R. (1990). Serum antibody response to antigens of oral gram-negative bacteria by cats with plasma cell gingivitis-pharyngitis. J. Dent. Res..

[236] Balzarini J., Vahlenkamp T., Egberink H., Hartmann K., Witvrouw M., Pannecouque C., Casara P., Nave J., De Clercq E. (1997). Antiretroviral activities of acyclic nucleoside phosphonates [9-(2-phosphonylmethoxyethyl) adenine, 9-(2-phosphonylmethoxyethyl) guanine, (R)-9-(2-phosphonylmethoxypropyl) adenine, and mdl 74,968] in cell cultures and murine sarcoma virus-infected newborn nmri mice. Antimicrob. Agents Chemother..

[237] Vahlenkamp T.W., De Ronde A., Balzarini J., Naesens L., De Clercq E., Van Eijk M., Horzinek M.C., Egberink H.F. (1995). (*R*)-9-(2-phosphonylmethoxypropyl)-2, 6-diaminopurine is a potent inhibitor of feline immunodeficiency virus infection. Antimicrob. Agents Chemother..

[238] Smith R.A., Remington K.M., Preston B.D., Schinazi R.F., North T.W. (1998). A novel point mutation at position 156 of reverse transcriptase from feline immunodeficiency virus confers resistance to the combination of (−)-β-2′, 3′-dideoxy-3′-thiacytidine and 3′-azido-3′-deoxythymidine. J. Virol..

[239] Hartmann K., Wooding A., Bergmann M. (2015). Efficacy of antiviral drugs against feline immunodeficiency virus. Vet. Sci..

[240] Schwartz A.M., McCrackin M.A., Schinazi R.F., Hill P.B., Vahlenkamp T.W., Tompkins M.B., Hartmann K. (2014). Antiviral efficacy of nine nucleoside reverse transcriptase inhibitors against feline immunodeficiency virus in feline peripheral blood mononuclear cells. Am. J. Vet. Res..

[241] De Rozieres S., Thompson J., Sundstrom M., Gruber J., Stump D.S., Aymeric P., VandeWoude S., Elder J.H. (2008). Replication properties of clade A/C chimeric feline immunodeficiency viruses and evaluation of infection kinetics in the domestic cat. J. Virol..

[242] Lee J.S., Mackie R.S., Harrison T., Shariat B., Kind T., Kehl T., Löchelt M., Boucher C., VandeWoude S. (2017). Targeted enrichment for pathogen detection and characterization in three felid species. J. Clin. Microbiol..

[243] Paulson J.N., Pop M., Bravo H. (2014). Metagenomeseq: Statistical analysis for sparse high-throughput sequencing. Bioconductor.

[244] Paulson J.N., Pop M., Bravo H. (2013). Metagenomeseq: Statistical analysis for sparse high-throughput sequencing. Bioconduct. Package.

[245] Hunt P.W., Deeks S.G., Rodriguez B., Valdez H., Shade S.B., Abrams D.I., Kitahata M.M., Krone M., Neilands T.B., Brand R.J. (2003). Continued CD4 cell count increases in HIV-infected adults experiencing 4 years of viral suppression on antiretroviral therapy. Aids.

[246] Leggott P.J. (1992). Oral manifestations of HIV infection in children. Oral Surg. Oral Med. Oral Pathol..

[247] Heron S.E., Elahi S. (2017). HIV infection and compromised mucosal immunity: Oral manifestations and systemic inflammation. Front. Immunol..

[248] Girard N., Servet E., Biourge V., Hennet P. (2009). Periodontal health status in a colony of 109 cats. J. Vet. Dent..

[249] Perry R., Tutt C. (2015). Periodontal disease in cats: Back to basics—With an eye on the future. J. Feline Med. Surg..

[250] Zahradnik R., Magnusson I., Walker C., McDonell E., Hillman C., Hillman J. (2009). Preliminary assessment of safety and effectiveness in humans of probiora3™, a probiotic mouthwash. J. Appl. Microbiol..

[251] Seminario-Amez M., López-López J., Estrugo-Devesa A., Ayuso-Montero R., Jané-Salas E. (2017). Probiotics and oral health: A systematic review. Med. Oral Patol. Oral Cir. Bucal.

[252] Teughels W., Durukan A., Ozcelik O., Pauwels M., Quirynen M., Haytac M.C. (2013). Clinical and microbiological effects of lactobacillus reuteri probiotics in the treatment of chronic periodontitis: A randomized placebo-controlled study. J. Clin. Periodontol..

[253] Galilee M., Alian A. (2018). The structure of FIV reverse transcriptase and its implications for non-nucleoside inhibitor resistance. PLoS Pathog..

[254] Doménech A., Miró G., Collado V.M., Ballesteros N., Sanjosé L., Escolar E., Martin S., Gomez-Lucia E. (2011). Use of recombinant interferon omega in feline retrovirosis: From theory to practice. Vet. Immunol. Immunopathol..

[255] Leal R.O., Gil S., Duarte A., McGahie D., Sepúlveda N., Niza M.M., Tavares L. (2015). Evaluation of viremia, proviral load and cytokine profile in naturally feline immunodeficiency virus infected cats treated with two different protocols of recombinant feline interferon omega. Res. Vet. Sci..

[256] Mari K., Maynard L., Sanquer A., Lebreux B., Eun H.M. (2004). Therapeutic effects of recombinant feline interferon-co on feline leukemia virus (FELV)-infected and felv/feline immunodeficiency virus (FIV)-coinfected symptomatic cats. J. Vet. Intern. Med..

[257] Künzi M.S., Pitha P.M. (1996). Role of interferon-stimulated gene ISG-15 in the interferon-ω-mediated inhibition of human immunodeficiency virus replication. J. Interferon Cytokine Res..

[258] Adolf G. (1995). Human interferon omega—A review. Mult. Scler. Houndmills Basingstoke Engl..

